# *Cryptococcus neoformans* urease affects the outcome of intracellular pathogenesis by modulating phagolysosomal pH

**DOI:** 10.1371/journal.ppat.1007144

**Published:** 2018-06-15

**Authors:** Man Shun Fu, Carolina Coelho, Carlos M. De Leon-Rodriguez, Diego C. P. Rossi, Emma Camacho, Eric H. Jung, Madhura Kulkarni, Arturo Casadevall

**Affiliations:** 1 Department of Molecular Microbiology and Immunology, Johns Hopkins Bloomberg School of Public Health, Baltimore, Maryland, United States of America; 2 Department of Microbiology and Immunology, Albert Einstein College of Medicine, Bronx, New York, United States of America; University of Birmingham, UNITED KINGDOM

## Abstract

*Cryptococcus neoformans* is a facultative intracellular pathogen and its interaction with macrophages is a key event determining the outcome of infection. Urease is a major virulence factor in *C*. *neoformans* but its role during macrophage interaction has not been characterized. Consequently, we analyzed the effect of urease on fungal-macrophage interaction using wild-type, urease-deficient and urease-complemented strains of *C*. *neoformans*. The frequency of non-lytic exocytosis events was reduced in the absence of urease. Urease-positive *C*. *neoformans* manifested reduced and delayed intracellular replication with fewer macrophages displaying phagolysosomal membrane permeabilization. The production of urease was associated with increased phagolysosomal pH, which in turn reduced growth of urease-positive *C*. *neoformans* inside macrophages. Interestingly, the *ure1* mutant strain grew slower in fungal growth medium which was buffered to neutral pH (pH 7.4). Mice inoculated with macrophages carrying urease-deficient *C*. *neoformans* had lower fungal burden in the brain than mice infected with macrophages carrying wild-type strain. In contrast, the absence of urease did not affect survival of yeast when interacting with amoebae. Because of the inability of the urease deletion mutant to grow on urea as a sole nitrogen source, we hypothesize urease plays a nutritional role involved in nitrogen acquisition in the environment. Taken together, our data demonstrate that urease affects fitness within the mammalian phagosome, promoting non-lytic exocytosis while delaying intracellular replication and thus reducing phagolysosomal membrane damage, events that could facilitate cryptococcal dissemination when transported inside macrophages. This system provides an example where an enzyme involved in nutrient acquisition modulates virulence during mammalian infection.

## Author summary

*Cryptococcus neoformans* is a relatively frequent cause of life-threatening infection in severely immunocompromised patients, especially those with AIDS. Persistence of infection involves residence within macrophages, where *C*. *neoformans* can survive and replicate while residing in the phagolysosome. New treatments may be developed from a better understanding of how this pathogen resists clearance from and adapts for persistence in host phagocytic cells. In this study, we demonstrate a novel role for urease, a major virulence factor of *C*. *neoformans*, in its interaction with macrophages. This enzyme is able to break down urea into ammonia, which is a base, thus raising the surrounding pH. In the context of a mammalian infection, we show that cryptococcal urease increases the phagolysosomal pH which delays yeast replication, therefore causing less damage to macrophages and prolongs intracellular residence. Moreover, urease promotes *C*. *neoformans* exit from macrophages without killing the host cells. Overall, our data implies that urease also contributes to virulence by allowing the pathogen to persist and disseminate in macrophages.

## Introduction

*C*. *neoformans*, a major life-threatening fungal pathogen predominantly infects severely immunocompromised patients and causes over 180,000 deaths per year worldwide [1]. *C*. *neoformans* is ubiquitous, although is most frequently found in soils contaminated with bird excreta or from trees [2–11]. Current treatments for Cryptococcosis often fail, are inadequate and/or unavailable for these infections, especially in developing countries. Therefore, it is important to study the fundamental pathogenic processes of *C*. *neoformans* to discover new treatments against this pathogen.

Human infection with *C*. *neoformans* follows inhalation of spore or yeast cells. In healthy individuals, pulmonary infections with *C*. *neoformans* are normally controlled and macrophages play a central role [12,13]. Soon after phagocytosis, the *Cryptococcus*-containing phagosome undergoes maturation, acidification and lysosome fusion [14–17]. However, *C*. *neoformans* is a facultative intracellular pathogen that it is able to survive and persist in mature phagolysosome, and can become latent and localized within the giant cells or macrophages in granulomas [15,18–22]. Depletion of macrophages is associated with improved survival of infected mice, supporting the notion that yeast cell cells are maintained within macrophages and as such, this host cell can constitute a niche for dissemination and persistence [23]. In the rat, latent infection resides in macrophages [18]. Infection can reactivate in conditions of weakened immunity, with intracellular replication and dissemination [15,17,20,22,24]. Consequently, the ability of *C*. *neoformans* to survive and replicate intracellularly contributes to different stages of cryptococcal pathogenesis [25–27]. It has been proposed that this intracellular pathogenic strategy emerged from interactions with amoebae in the environment [28–30]. A recent study reports that *C*. *neoformans* spends a relatively short time (~80 min) inside *Dictyostelium discoideum* and is expulsed before yeast replication occurs [30].

*C*. *neoformans* expresses virulence factors that promote its pathogenicity, including formation and enlargement of a polysaccharide capsule, melanin production, extracellular secretion of various enzymes including phospholipase, urease, etc. The role of capsule and melanin in macrophage-pathogen interaction are well understood. The capsule interferes with phagocytosis, by potentially masking macrophage receptor binding sites and polysaccharide shed by the yeast is immunosuppressive [31–34]. Moreover, both capsule and melanin protect *C*. *neoformans* from intracellular killing by providing protection against reactive oxygen species (ROS) as well as antimicrobial peptides [35,36]. However, mechanism by which urease contributes to intracellular pathogenesis is unknown.

Urease functions as a general virulence factor for many bacterial pathogens, such as *Helicobacter pylori* [37], and fungal pathogens *Cryptococcus* spp. and *Coccidioides posadasii* [38–40]. Urease catalyzes the hydrolysis of urea into carbon dioxide and ammonia [41,42]. Ammonia generated from ureolytic activity can serve as a nitrogen source. Since urea is evenly distributed throughout the human body it is conceivable it is used as a nutrient by mammalian pathogens [43]. Beyond its nutritional role, ureolytic activity enhances the invasion of *C*. *neoformans* to the central nervous system by promoting the yeast sequestration within the microcapillary beds of blood-brain barrier. The underlying mechanism is not known, but was hypothesized that ammonia generated by urease activity was toxic to microvascular endothelial cells [44–46]. Urease-mediated ammonia can also neutralize any acidic microenvironment and thus help pathogens to survive harsh pH of the phagolysosome. The neutralizing effect of *H*. *pylori*’s urease is well established, enabling that bacterium to colonize gastric mucosa [47,48]. In addition to its role in gastric colonization, *H*. *pylori* urease regulates the host-macrophage interaction by retarding the opsonization of *H*. *pylori* [49]. The enzyme can also modulate phagosomal pH and disrupt phagosome maturation to enhance the intracellular survival of *H*. *pylori* in macrophages [50]. Furthermore, it induces the expression of inducible NO-synthesizing enzyme (iNOS), a M1 macrophage polarization marker, in mouse macrophages [51]. In contrast, in both *C*. *neoformans* and *C*. *posadasii*, urease-producing strains promote the polarization of immune responses to a nonprotective Type 2 (T2) rather than a fungicidal Type 1 (T1) immune response [38,52]. Hence, bacterial and fungal urease may have different effects on macrophage activation, and the role of cryptococcal urease during macrophage-pathogen interaction, which may affect the appropriate immune response, is unexplored.

In this paper, we evaluated the role of urease on intracellular pathogenesis of *C*. *neoformans* in both amoebae and macrophages. We studied the effect of urease on the macrophage response to *C*. *neoformans*, as measured by host cell lysis and non-lytic exocytosis, cryptococcal replication inside macrophages and phagolysosomal pH. The results indicate that *C*. *neoformans* urease affects the non-lytic exocytosis and intracellular replication of the yeast by modulating phagolysosomal pH thus illustrating a new mechanism of by which this enzyme contributes to virulence.

## Results

### Urease and the interaction between *Acanthamoeba castellanii* and *C*. *neoformans*

**Fig 1:**
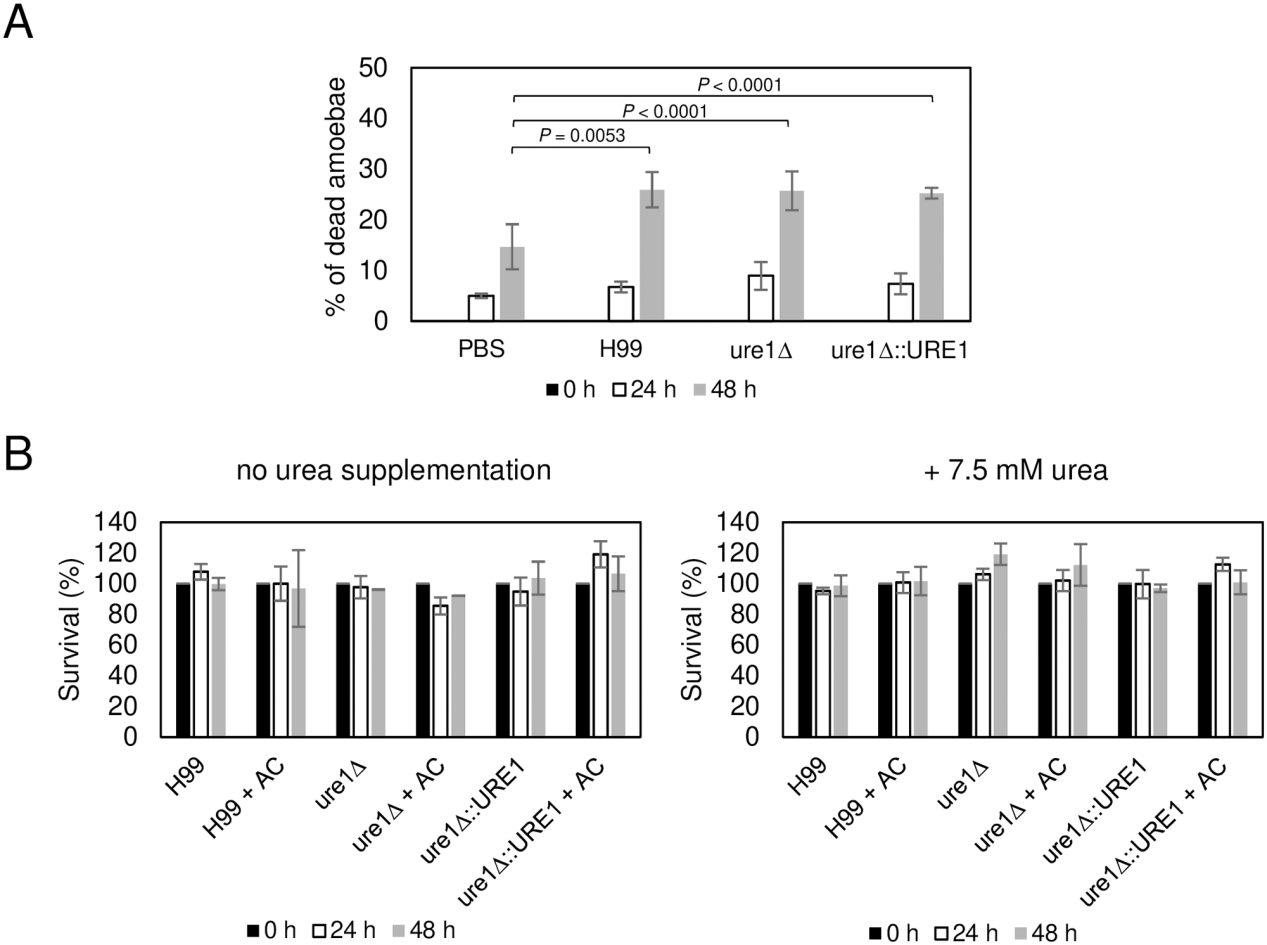
Urease has no effect on the survival of *C*. *neoformans* and killing of *A*. *castellanii* during co-incubation of *C*. *neoformans* and *A*. *castellanii*. *A*. *castellanii* (AC) was co-cultured with H99, *ure1*Δ or *ure1*Δ::*URE1* strain in DPBS with or without 7.5 mM urea. (A) The viability of *A*. *castellanii* was determined by Trypan Blue exclusion assay. The percentage of dead *A*. *castellanii* is determine by counting the number of Trypan Blue staining cells per total cell number counted. Two independent biological experiments were performed. Error bars are SD. *P* values shown in graph was calculated by Fisher’s exact test when each strain was compared with PBS alone at 48 h co-incubation. *P* > 0.05 when H99 and *ure1*Δ::*URE1* were compared with *ure1*Δ at 24 and 48 h by Fisher’s exact test. (B) The survival of cryptococcal strains were determined by colony form unit (CFU) after 0, 24 and 48 h. Two independent biological experiments were performed. Error bars are standard deviation (SD). *P* > 0.05 by Student’s *t* test.

Many virulence factors used by *C*. *neoformans* for survival in mammalian host such as capsule, melanin and phospholipase B1 are also important for the survival of *C*. *neoformans* in its natural environment, where it is subject to predation by amoebae [28,53]. The capacity of *C*. *neoformans* for mammalian virulence was proposed to result from the fortuitous selection of traits that allow survival in animal hosts by environmental predators [30,54]. To explore whether urease plays a role in the virulence towards amoebae we examined the viability of *A*. *castellanii* during the co-incubation with *C*. *neoformans*. Consistent to previous studies [28,55], the percentage of dead *A*. *castellanii* cells in the presence of *C*. *neoformans* (25–26%) was significantly higher than in PBS alone (14.7%) after 48 h co-incubation (Fig 1A). However, the percentage of *A*. *castellanii* cells killed by cryptococcal urease-positive and negative strains was similar (Fig 1A). We also examined the survival of *C*. *neoformans* with or without urease during co-incubation with *A*. *castellanii*. In addition, the buffer solution was supplemented with 7.5 mM urea to test whether the process of ureolysis could improve the survival of *C*. *neoformans* during the interaction with *A*. *castellanii*. However, no significant difference in survival between strains with urease and without urease was observed in all the tested conditions (Fig 1B). These results suggest that urease, which is a virulence factor for mammalian hosts, is not necessary for virulence in amoebae.

### Urease did not affect phagocytosis, phagosomal maturation and nitric oxide production

Macrophages play a central role in host response to cryptococcal infection and harbor the organism as an intracellular pathogen during latent infection. To investigate the effect of urease in intracellular pathogenesis we studied the response of macrophage when they were infected with either *C*. *neoformans ure1* deletion strain, its parental H99 or the *URE1* complemented strains. Studies have shown that *H*. *pylori* urease can affect phagocytosis [49], modulate the recruitment of lysosomal marker LAMP-1 to phagosome and thus prevent phagosomal maturation [50], and stimulates the expression of iNOS to induce nitric oxide generation production in mouse macrophages [51]. Therefore, we first measured the efficiency of phagocytosis of antibody-opsonized cells for the three strains by calculating the phagocytic index after incubation for 2 h (S1A Fig). We then tested if cryptococcal urease can affect the recruitment of LAMP-1 to phagosomes by examining percentage of LAMP-1 positive phagosomes (S1B Fig). To study if cryptococcal urease affects host iNOS expression, we infected macrophages with urease-positive and negative strains and measured the concentration of nitrite, a stable oxidation product of nitric oxide, in the culture supernatant using Griess assay (S1C Fig). Our data shows that cryptococcal urease does not affect the efficiency of antibody-mediated phagocytosis, phagosomal maturation, and nitric oxide production of macrophages during the infection. We also determined the ability of wild-type, urease deletion and complemented strains to survive intracellularly by enumerating the colony forming units (CFU). Our result show that all strains had similar intracellular survival in macrophages after 2 h phagocytosis (S1D Fig).

### Urease and non-lytic exocytosis

**Fig 2:**
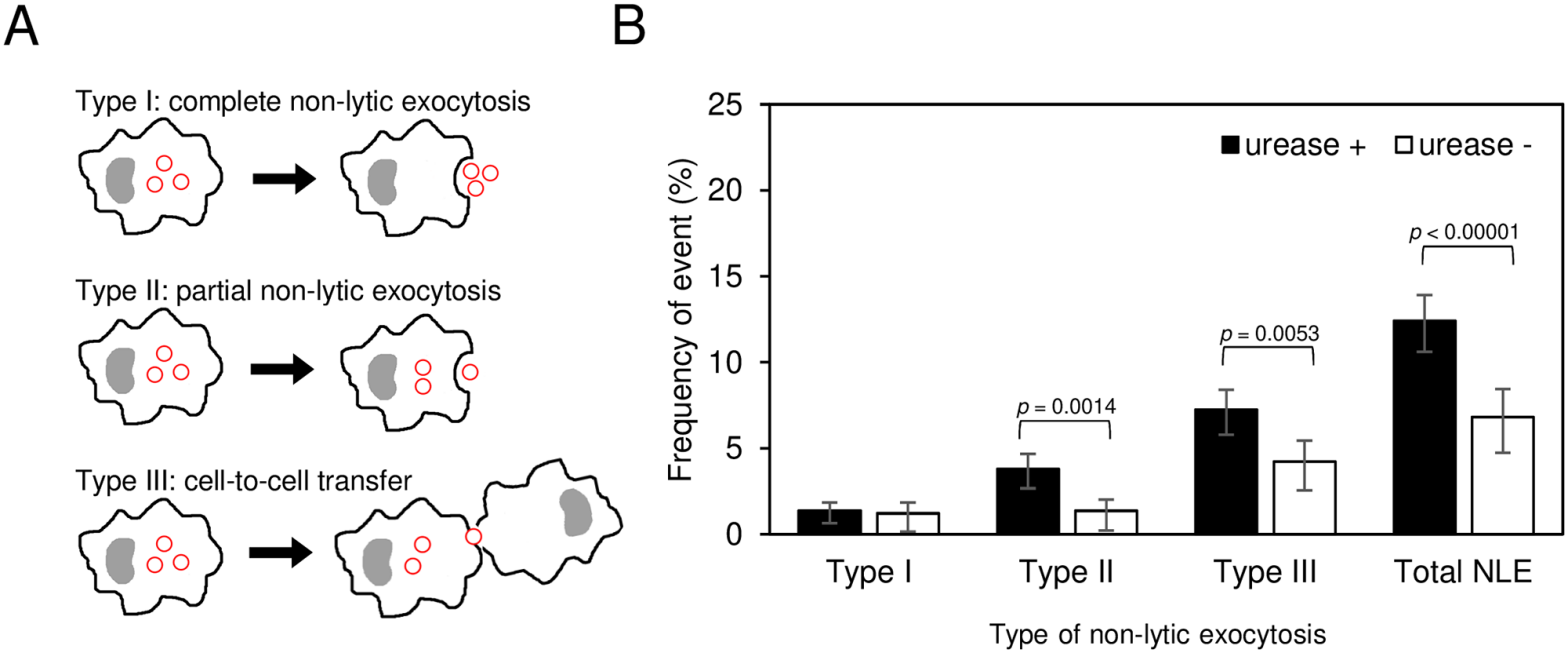
Urease affects non-lytic exocytosis (NLE). (A) Schematic representation shows types of NLE of *C*. *neoformans* from macrophages. The red circles represent *C*. *neoformans*. (B) BMDM were infected with urease-positive *C*. *neoformans* (H99 and *ure1*Δ::*URE1*) and urease-negative *C*. *neoformans* (*ure1*Δ). The frequency of NLE events was determined from 24 h time-lapse movies. Each strain was tested at least five times independently. Error bars represent 95% confidence interval of the mean. *P* values by Fisher’s exact test.

We studied host cell outcomes for infected macrophages with regards to non-lytic exocytosis. Three subcategories of non-lytic exocytosis, as defined on a previous study [56] were used: complete non-lytic exocytosis (type I), partial non-lytic exocytosis (type II) and cell-cell transfer (type III) (Fig 2A). Macrophages infected with *ure1*Δ mutant underwent fewer non-lytic exocytosis than those infected with urease producing *C*. *neoformans*, in particular partial non-lytic exocytosis (type II) and cell-cell transfer (type III) (Fig 2B). This result implies that the presence of urease has an effect on non-lytic exocytosis.

### Urea and host cell egress of *C*. *neoformans*

**Fig 3:**
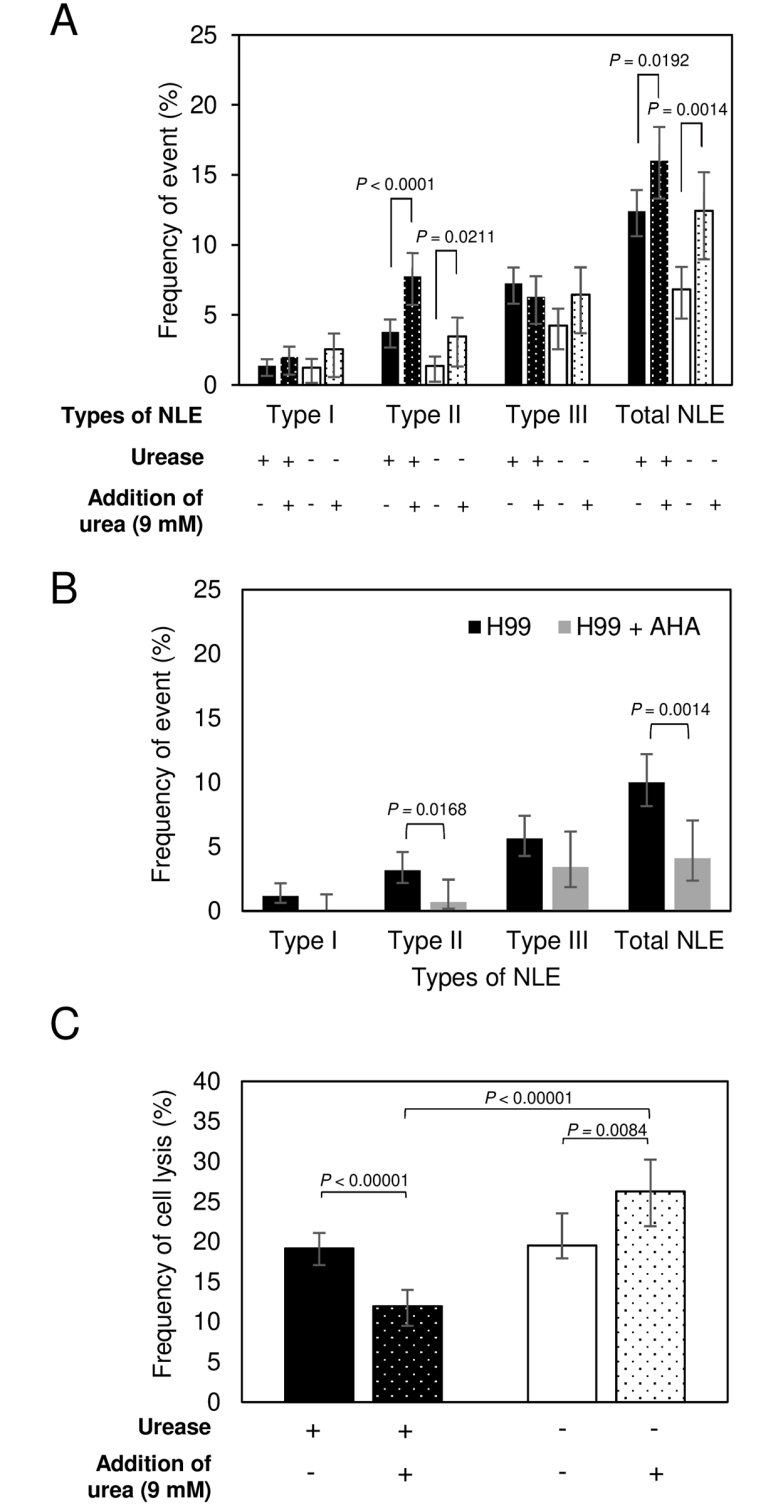
Urea supplementation affects non-lytic exocytosis. The frequency of (A-B) NLE event (Type I, complete NLE; type II, partial NLE; type III, cell-to-cell transfer), and (C) cell lysis was determined from 24 h time-lapse movies of BMDM infected with urease-positive strain (H99 or/and *ure1*Δ::*URE1*) and urease-negative strain (*ure1*Δ) in medium supplemented with or without 9 mM urea or 5 mM of urease inhibitor AHA. Each strain was tested five times independently in condition of no treatment, three times with urea supplementation and two times in the presence of AHA. Error bars represent 95% confidence interval of the mean. *P* values by Fisher’s exact test.

We hypothesized that if ureolytic activity of urease influenced non-lytic exocytosis, that the addition of urea to the media would also affect the frequency of these events. Consequently, we adjusted the concentration of urea in cell media to 9 mM, which is the level found in plasma from mouse [57], and studied the interaction of *C*. *neoformans* and macrophages. Total non-lytic exocytosis events remained higher with urease-positive strains and increased by approximately 23% with increasing concentration of urea (Fig 3A). However, the increase in exocytosis was also noted when macrophages were infected with urease deleted strain (Fig 3A). Hence, urea appeared to affect the frequency of non-lytic exocytosis independently of any effect related to the urea hydrolysis by urease, precluding definitive conclusions.

To investigate whether urease enzymatic activity affected non-lytic exocytosis, we infected BMDM with H99 in the presence of 5 mM of the urease inhibitor acetohydroxamic acid (AHA), a concentration that inhibits 50% of yeast urease activity while presenting minimal toxicity to murine macrophages (S2 Fig), and measured the frequency of non-lytic exocytosis. Addition of AHA decreased non-lytic exocytosis to a level comparable to that observed with the urease deletion mutant (Fig 3A and 3B). This result is consistent with and supports the notion that urease mediated urea hydrolysis modulates the frequency of non-lytic exocytosis.

We observed no significant difference in the frequency of host cell lysis in the presence versus absence of urease when there is no urea supplementation (Fig 3C). However, after the concentration of urea in the culture medium was adjusted to 9 mM cell lysis events of macrophages infected with urease-positive strain decreased by 38%, whereas the event of cell lysis with urease-negative strain increased by 35% (Fig 3C). That in turn led to the significant difference of cell lysis between macrophages containing urease-positive strain and urease-negative strain (Fig 3C).

### Urease and intracellular growth and survival

**Fig 4:**
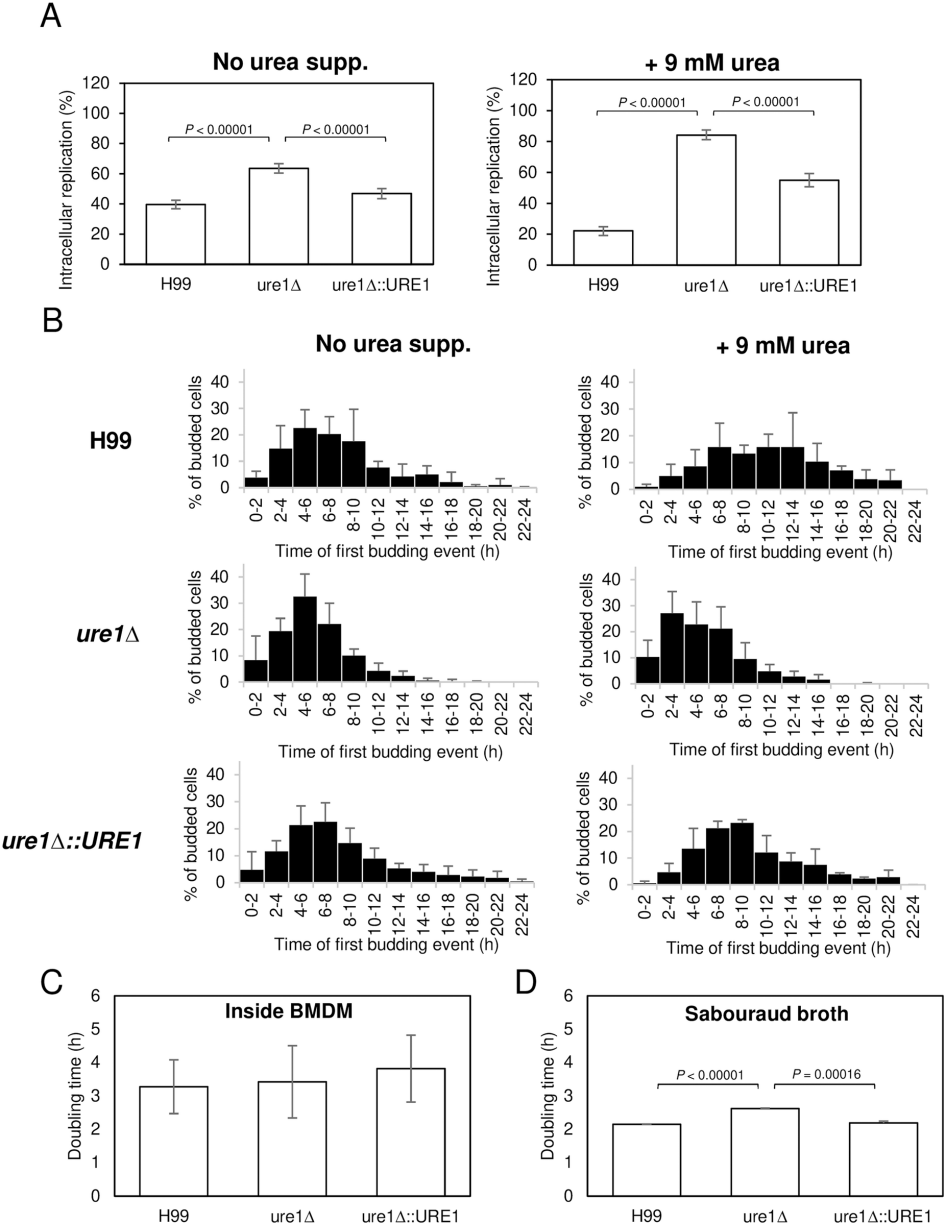
Urease affects intracellular replication. (A) More cryptococcal cells undergo intracellular replication in the absence of urease. BMDM were infected with H99, *ure1*Δ or *ure1*Δ::*URE1* strain in the medium supplemented with 9 mM urea (right panel) or no urea supplementation (left panel). Number of cells which undergoes intracellular replication were counted in 24 h time-lapse movies. Each strain was tested five times independently in conditions of no urea supplementation or three times with urea supplementation. Error bars represent 95% confidence interval of the mean. *P* values by Fisher’s exact test. (B) Cryptococcal cells with urease have a delayed replication inside macrophage. The histogram represents the percentage of cells which undergo first budding at indicated times after phagocytosis. Each strain was tested five times independently in conditions of no urea supplementation or three times with urea supplementation. Error bars are SD. (C) Urease does not affect the doubling time of cryptococcal intracellular replication. The time interval between the first and second budding of intracellular cryptococcal strains was measured during the 24 h time-lapse movies. Each strain was tested at least five times independently. Error bars are SD. *P* = 0.83569 when H99 were compared with *ure1*Δ; *P* = 0.61023 when *ure1*Δ::*URE1* were compared with *ure1*Δ by Student’s *t* test. (D) Doubling time was determined from growth curve among strains when they are cultured in Sabouraud broth at 30 °C with shaking for up to 72 h. Each strain was tested in triplicate. Error bars are SD. *P* value by Student’s *t* test.

We compared the intracellular replication of cryptococcal strains with or without urease. For each strain, we analyzed more than 800 internalized cryptococcal cells in five 24-hour time-lapse movies for their ability to replicate intracellularly. In wildtype *C*. *neoformans*, 39.6% of cells underwent replication inside macrophages while 63.5% occurred for *ure1*Δ cells, i.e., *ure1*Δ cells replicated nearly twice as more than wildtype (Fig 4A). Adding 9 mM urea to the medium resulted in larger difference in intracellular replication between wild-type and urease deletion mutant, with decreased number of replication on H99 and increased number of replication on *ure1*Δ (Fig 4A). We also investigated whether the presence of urease affected the onset of intracellular replication, and we measured the time of first budding after the cells were phagocytized by macrophages. Urease-positive strains had more cells that started replication later than urease-negative cells although both of the peaks are at 4–6 h after phagocytosis (Fig 4B). Therefore, even among those cells that replicated inside macrophages, urease-positive strains manifested slightly delayed replication compared to *ure1*Δ strain. The delay became more pronounced when urea (9 mM) was supplemented to the medium, resulting in a peak shift of urease-positive cells from 4–6 h to 8–10 h, implying that cells took longer to begin to replicate in this condition (Fig 4B). Collectively, these data demonstrate that urease ureolytic activity is strongly linked to the delayed onset of cell replication. However, once the cells started to replicate, the doubling time of all strains was very similar (Fig 4C), although *ure1*Δ strain had approximately 30 min longer doubling time in a standard laboratory condition (Sabouraud broth with shaking at 30 °C) when comparing to H99 and the complemented strain (Fig 4D).

A prior study had shown that prolonged cell cycle progression resulted in cells with larger capsule, which was associated with protection during phagocytosis and enhance intracellular survival [58]. Since urease-positive strains manifested delayed intracellular replication, we investigated whether urease-positive strains had larger capsules after phagocytosis. Consequently, we measured the capsule size of H99, urease deletion and complemented strains harvested from macrophages after 16 h infection. There was no significant difference in capsule size between urease-positive and negative strains inside macrophages (S3 Fig).

### Lysosomal permeability for urease-positive and negative strains

**Fig 5:**
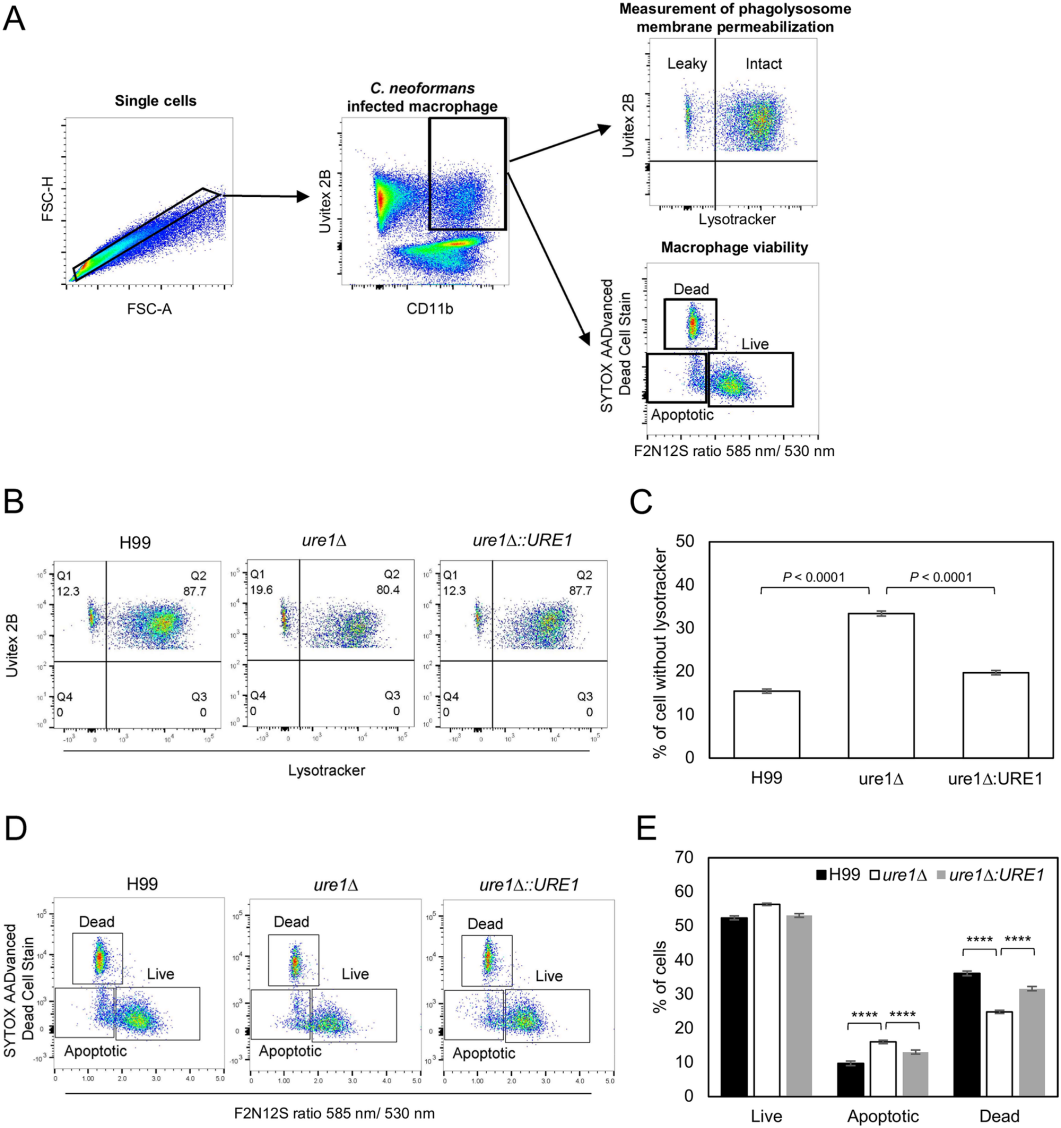
Macrophages infected with urease-positive strain have less phagolysosomal membrane permeabilization (PMP). (A) Gating strategy for analyzing PMP and macrophage viability. Single cells were first identified using FSC-A and FSC-H. CD11b and Uvitex 2B double positive cells were then gated and defined as *C*. *neoformans* infected macrophages. Frequency of PMP of these infected macrophages were measured using Lysotracker deep red staining. Macrophage viability were also determined by using SYTOX and F2N12S staining. (B) Representative flow cytometry dot plots shows comparison of the frequency of macrophages which were infected with H99, *ure1* and *ure1*Δ::*URE1* for 24 h with permeable phagosomes as determined by loss of Lysotracker deep red staining. The percentage of macrophage which retains Lysotracker deep red (Q2 population) and losses Lysotracker deep red (Q1 population) are shown. (C) Quantification of loss of Lysotracker deep red staining (Q1 population) is shown. Data shown is mean from two independent biological experiments. Error bars represent 95% confidence interval of the mean. *P* values by Fisher’s exact test. (D-E). Flow cytometry dot plots and bar chart shows the comparison of live, apoptotic and dead macrophages which are infected with H99, *ure1* and *ure1*Δ::*URE1* for 24 h. Data shown is mean from two independent biological experiments. Error bars represent 95% confidence interval of the mean. *P* values by Fisher’s exact test.

The intracellular replication of *C*. *neoformans* is tightly correlated to lysosomal damage, such that macrophages with higher numbers of cryptococcal cells manifest greater lysosomal membrane permeabilization [59]. Consequently, we investigated whether lysosomal membrane permeabilization was associated with the urease activity for *C*. *neoformans* strains. We stained macrophages with Lysotracker Deep Red, which localizes to and labels acidic organelle such that loss of fluorescence signal indicates lysosomal damage. The number of cells manifesting loss of Lysotracker fluorescence was then quantified by flow cytometry after 24 h of infection. *C*. *neoformans* infected macrophages developed loss of Lysotracker signal (Q1 population in Fig 5A and 5B). As negative control, macrophages infected with heat killed H99 manifested no loss of Lysotracker fluorescence [60]. The percentage of Lysotracker-loss macrophages infected with H99, *ure1* deletion and complemented strains was highest for *ure1*Δ-infected macrophages (Fig 5C). This result suggests that host cells which are infected with *ure1* deletion strain undergo significantly more lysosomal membrane damage.

We further investigated if different degrees of lysosomal damage were associated with the presence or absence of urease resulted in different degree of apoptosis given that lysosomal damage can release cathepsins into the cytosol and trigger programmed cell death [61]. Therefore, we stained macrophages with SYTOX as an indicator of death cells and F2N12S (dye that indicates membrane potential) to distinguish apoptotic from healthy cells (Fig 5A). There was no significant difference in the percentage of live cells in the sets infected with H99, *ure1* deletion and complemented strains respectively (Fig 5D and 5E). However, there was a slight difference of the proportion of apoptotic and death cells between urease-positive strains and urease deletion mutant (Fig 5D and 5E). These results suggest that the presence or absence of urease translates in different intracellular growth rates with the absence of urease being associated with greater phagolysosomal membrane permeabilization, and a shift on how macrophage death occurs.

### Urease increased phagolysosomal pH

**Fig 6:**
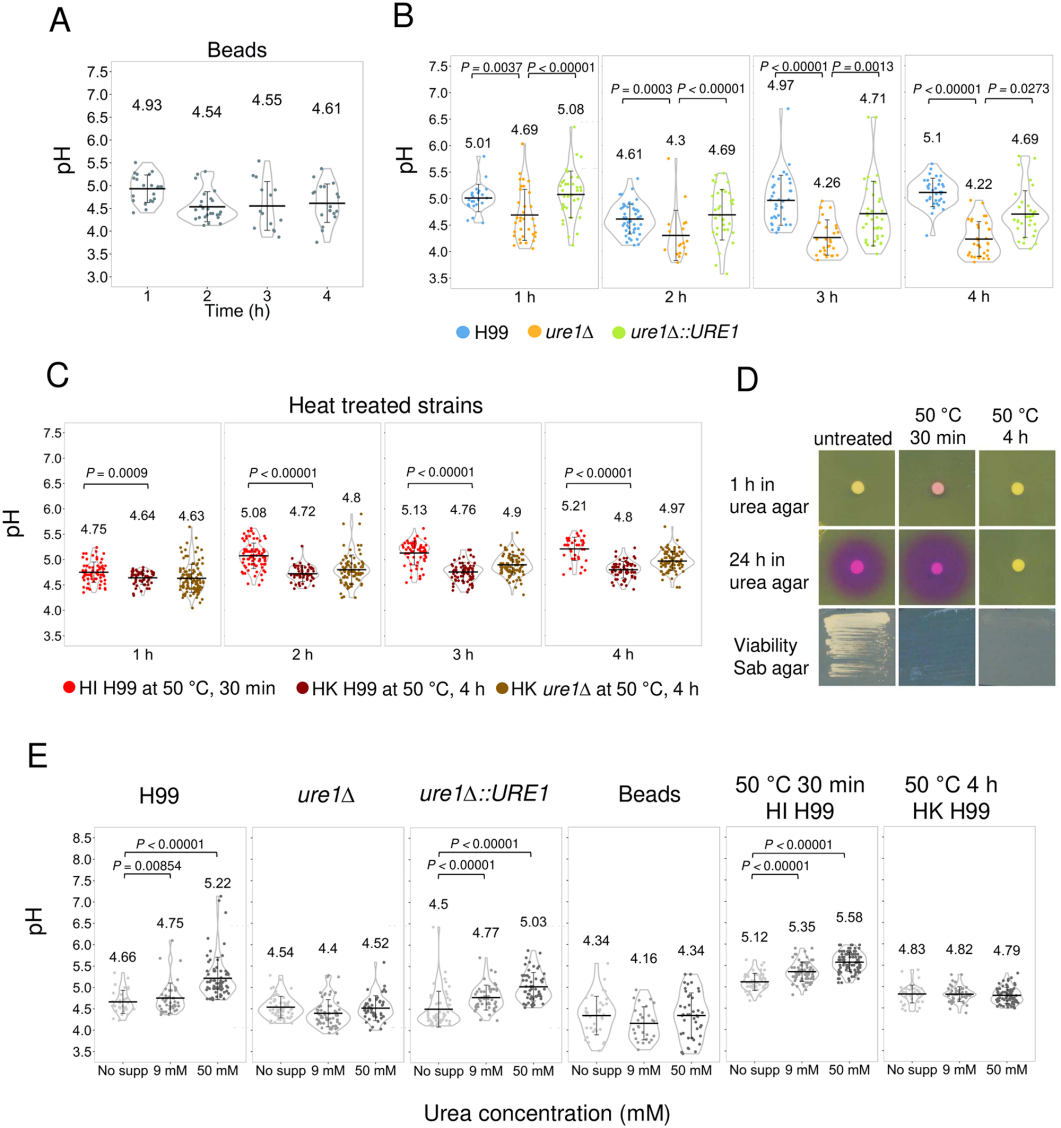
The presence of urease increases the phagolysosomal pH. (A–C) BMDM were infected with Oregon green labelled (A) IgG-coated polystyrene beads (B) H99, *ure1*Δ or *ure1*Δ::*URE1*, and (C) heat-inactivated (HI) H99 (heat inactivated at 50 °C for 30 min), heat-killed (HK) H99 or *ure1*Δ (heat killed at 50 °C for 4 h). Phagolysosomal pH was measured by using dual-excitation ratio fluorescence imaging at indicated time point. Each dot represents pH of individual phagolysosomes. Violin plot displays the probability density of dataset with means (middle bar) and SD. Data in A-C are from one representative experiment (from three biologically independent experiments). An additional phagolysosomal pH data with H99, *ure1*Δ or *ure1*Δ::*URE1* and summary plots which represent the average of individual phagolysosomes pH in each biological independent experiment are shown in S4 Fig. *P* values by Mann-Whitney *U* test. (D) Untreated and heat treated H99 were spotted on Christensen’s urea agar and incubated at 30 °C for 1 h and 24 h to detect their urease activity. Cells were also streaked on Sabouraud (Sab) agar and incubated at 30 °C for 48 h to test their viability. (E) The phagolysosomal pH was measured in BMDM infected with H99, *ure1*Δ, *ure1*Δ::*URE1*, IgG coated polystyrene bead, HI H99 (heat inactivated at 50 °C for 30 min) or HK H99 (heat killed at 50 °C for 4 h) in the medium with no additional urea supplement (no supp) or supplemented with 9 mM and 50 mM urea at 4 h. Data were obtained from two independent experiments but shown from one representative experiment. Summary plot of replicates are shown in S5A Fig. *P* values by Mann-Whitney *U* test.

*C*. *neoformans* urease breaks down urea into ammonia and carbon dioxide, and subsequently ammonia reacts with water to produce hydroxyl ions that increase pH. A previous study shows that non-lytic exocytosis is influenced by phagolysosomal pH [62]. In addition, *C*. *neoformans* does not grow well in alkaline pH [14]. We hypothesized that the presence of urease would increase phagolysosomal pH, and the alterations in pH would then increase the frequency of non-lytic exocytosis events and affect cryptococcal growth [62,63]. To test this hypothesis, we measured and compared the phagolysosomal pH with the urease-positive or negative strains. Unlike prior studies, we devised a method to measure pH in specific *C*. *neoformans*-containing phagolysosomes by conjugating a pH sensitive probe to 18B7 antibody, which binds to cryptococcal capsule [59]. To validate our methodology, we measured phagolysosomal pH associated with the ingestion of Oregon green conjugated 18B7 labeled polystyrene beads, which have been widely used to study phagocytosis in macrophages. Absolute pH was calculated using a pH standard curve obtained from the measurements of pH of phagosomes containing beads (S4A Fig and Materials and methods). The data showed that the acidification started rapidly and by 2 h phagolysosomes had reached the lowest pH (mean = pH 4.5), which remained constant until 4 h (Fig 6A and S4B Fig). Previous study has shown that the bead-containing phagolysosomes reach pH = 5 in 15 min and up to 30 min [64], which is consistent with our result showing average pH of 4.9 in bead-containing phagolysosomes in the first hour after ingestion. We proceeded to measure the pH of phagolysosome containing wild-type, *ure1* deletion and complement strains. Phagolysosomes containing wild-type cells had a pH ranging from 4.6 to 5.1, which was consistently higher than those containing urease deletion mutant cells (pH ranging from 4.2 to 4.7) through all the infection periods measured (Fig 6B, S4C and S4D Fig). Our result is consistent with a prior study showing that the pH of phagolysosomes containing live cryptococcal cells is 4.7 after 3 h infection using a different methodology [14]. The phagolysosomes containing the cells from the urease complement strain had a similar pH (4.7–5.1) as those containing wild-type cells and constantly had higher pH than cells deficient in urease at the first two hours of phagocytosis, but the results were inconsistent with those containing wild-type for longer time intervals (Fig 6B and S4C Fig). This finding could result from differences in urease level expression in the complemented strain relative to wild-type expression and/or other uncharacterized factors affecting the reconstituted strain. We also observed considerable pH variation among individual phagolysosomes, which could reflect many factors including differences in the timing of phagocytosis, heterogeneous microenvironments or the cellular position of the phagolysosomes (peripheral vs juxtanuclear) [65] or plain stochastic variation. Overall, phagolysosomes containing both wild-type and urease complemented strains had higher pH than the bead- and *ure1*Δ strain-containing phagosomes in the first two hours after phagocytosis, consistent with a mechanism whereby urease increases phagolysosomal pH through hydrolysis of urea, which is present in the system from the metabolism of macrophages and from fetal calf serum in the macrophage media.

We also determined the pH of phagolysosome containing heat-inactivated H99 (50 °C for 30 min). Although we expected that it would behave comparably pH of phagolysosomes containing polystyrene beads, the pH was significantly higher (pH 5.1) from 2–4 h (Fig 6C and S4E Fig). Surprisingly, we found that urease activity was not abolished by heat-inactivating to 50 °C for 30 min, since the plating of heat-inactivated cells on Christensen urea agar turned pink (Fig 6D). Of note, the color effect in urea agar was faster with heat-inactivated cells than with alive cells, suggesting that the heating may have liberated the enzyme. Given this result we repeated the experiment with H99 cells killed by heating to 50 °C for a longer period of time (4 h) which was effective in inactivating the urease activity (Fig 6C). The phagolysosomal pH indeed became lower (pH 4.7) after macrophages ingested H99 heat killed at 50 °C for 4 h when compared to 30 min (Fig 6C and S4E Fig). We note that phagolysosomal pH of heat-killed H99 at 50 °C for 4 h was not as low as the pH of phagolysosome containing beads or the urease deletion mutant. A similar phagosomal pH value was observed after ingestion heat-killed urease deficient cells (50 °C for 4 h) (Fig 6C and S4E Fig), suggesting that part of the increase in pH is independent of the presence of urease. We hypothesize these components could derive from leakage of intracellular contents including proteins with functional groups such as amino acids and carboxylic groups that can absorb hydronium ions, and buffer the acid flux such that it does not reach the low pH observed with polystyrene beads. However, the data with urease deficient mutant and urease inactivation by heat killing demonstrates that cryptococcal urease contributes to neutralize and therefore increase the phagosomal pH after ingestion by murine macrophages.

To confirm whether the increase of pH was a result of urease activity, we supplemented the media with urea at different concentrations (9 mM or 50 mM). To establish that urea can freely pass across cell membrane, we measured urea in macrophages using a colorimetric assay. Incubation of macrophages with urea raised their urea content to physiological urea concentration of 9 mM (S5A Fig). Urea supplementation was associated with a more alkaline phagolysosomal pH in macrophages containing wild-type and urease complemented strains as well as heat-inactivated H99 (50 °C for 30 min), but not urease deletion strain, bead or heat killed H99 (50 °C for 4 h) (Fig 5E and S5B Fig). These results suggest that the increased phagolysosomal pH is associated with urease degradation of available urea.

### *C*. *neoformans* growth is affected by pH

**Fig 7:**
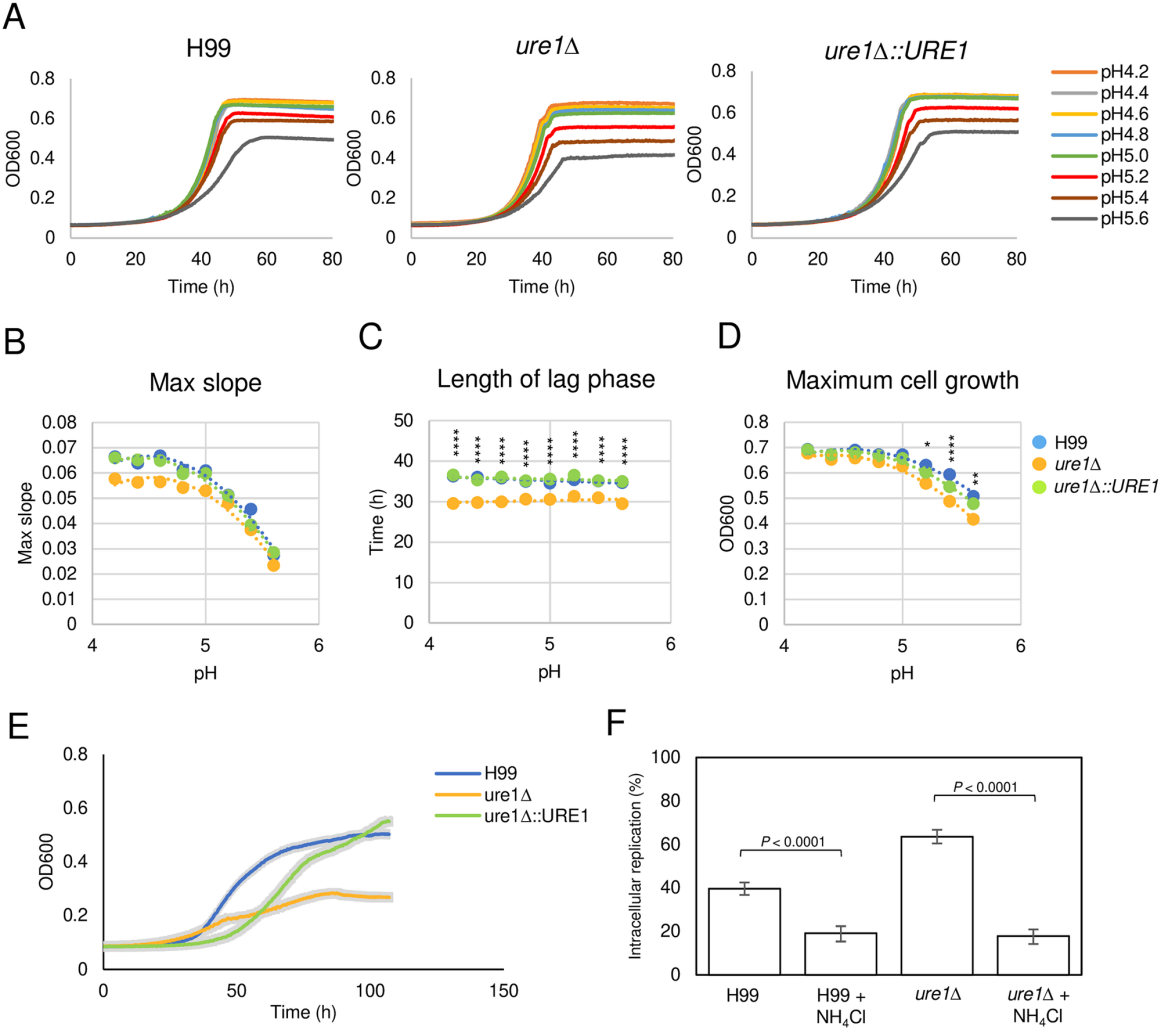
Cryptococcal cell growth is affected by pH. (A) Growth curves of H99, *ure1*Δ, *ure1*Δ::*URE1* strains were performed in buffered minimal medium with pH ranging from 4.2 to 5.6 at 30 °C for 3 days. Shown is average from three independent experiments. (B) The maximum slopes (two-way analysis of variance [ANOVA]: strains, *P* = 0.08022; pH, *P* < 0.0001), (C) the length of lag phase (two-way ANOVA: strains, *P* < 0.0001; pH, *P* = 0.17866; with Tukey’s multiple-comparison test *P* < 0.0001) and (D) the maximum cell growth (two-way ANOVA: strains, *P* < 0.0001; pH, *P* < 0.0001; with Tukey’s multiple-comparison test ****P < 0.0001, ** *P* = 0.0341, * *P* = 0.0017) were determined from the growth curves and plotted against corresponding pH. (E) Growth curves of H99, *ure1*Δ, *ure1*Δ::*URE1* strains were performed in buffered minimal medium with pH 7.4 at 30 °C for 4 days. Shown is average from three independent experiments. The shaded area represents SD. (F) Intracellular replication upon supplementation with ammonium chloride. The number of cells that underwent intracellular replication were counted in 24 h time-lapse movies. Each strain was tested five times independently without NH_4_Cl treatment and three times with NH_4_Cl treatment. Error bars represent 95% confidence interval of the mean. *P* values by Fisher’s exact test.

To investigate how pH could affect the intracellular growth results we studied the growth of *C*. *neoforman*s as a function of the pH. We used the growth curves to determine three characteristic growth values i.e. growth rate represented by the maximum slope, length of lag phase, and the maximum cell growth and compared them among strains (Fig 7A and 7B). Our experiment displayed an inverse relationship between the growth rate of all tested strains and increasing pH. The growth rate decreased approximately 3-fold from pH 4.2 to 5.6. Hence, *C*. *neoformans* grew best in the most acidic milieu, a finding that combined with phagolysosomal pH measured for urease sufficient and deficient strains suggests that the effect of urease on cryptococcal intracellular replication is due to its effect in neutralizing pH since pH affects yeast growth rate.

**Fig 8:**
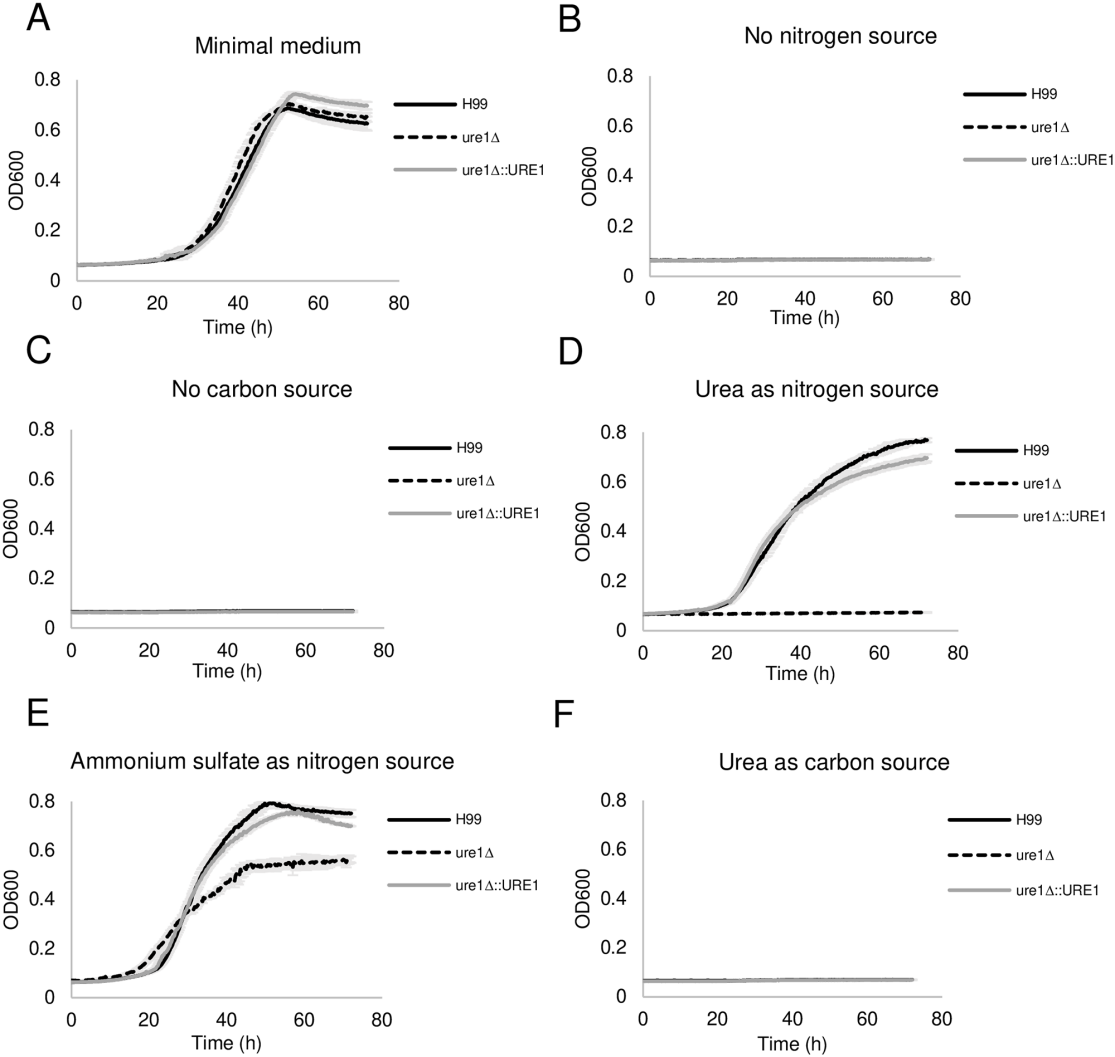
*C*. *neoformans* utilizes urea as a nitrogen source, but not as a carbon source, in the presence of urease. Growth curves of H99, *ure1*Δ and *ure1*Δ::*URE1* strains were performed at medium with (A) glycine as sole nitrogen source, (B) no nitrogen source, (C) no carbon source, (D) urea as sole nitrogen source, (E) ammonium sulfate as nitrogen source, and (F) urea as sole carbon source. The data average from three independent experiments. Error bars indicate SD.

The strain *ure1* Δ had shorter lag phase than H99 and *URE1*-complemented strains at all pH tested (Fig 7C). One possible explanation is that there is a metabolic cost of producing the enzyme, especially when adjusting to the nutrient-limited medium we used in this particular experiment. However, once the urease-positive strains adapt to the environment, the rate of growth can return to maximum. Interestingly, the maximum cell growth of *ure1*Δ was similar to that of H99 and *URE1*-complemented strains in acidic pH, but gradually decreased closer to neutral pH (Fig 7D). Therefore, we questioned if the mammalian physiological pH affected the growth of *ure1*Δ, so the growth curves of H99, *ure1*Δ and *URE1*-complemented strains were determined in minimal medium buffered at pH 7.4. The result showed that *ure1*Δ had a severe growth defect at pH 7.4 (Fig 7E) while all strains grow equally in unbuffered minimal medium (Fig 8A), suggesting that urease is required for neutral and alkaline tolerance.

To study further the association of phagolysosomal pH and the intracellular replication of *C*. *neoformans*, we added the weak base ammonium chloride to the medium, which is known to neutralize phagosomal acidity and inhibit cryptococcal intracellular growth [14,62,66], and measured intracellular replication of urease deletion mutant in a 24 h time-lapse movie. The addition of ammonium chloride retarded intracellular replication of *C*. *neoformans* independent of the presence of urease (Fig 7F), providing strong support for the notion that the higher phagosomal pH was the cause of the retarded cryptococcal intracellular replication.

### Utilization of urea by *C*. *neoformans*

Urease is not only able to elevate the pH of microenvironment, but it also serves as a nitrogen source for pathogenic microbes such as *Actinomyces naeslundii* and *Bacillus cereus* during infection by hydrolyzing urea to ammonia [67–69]. A prior study showed that urease activity is required for cryptococcal growth in agar medium with urea as the only nitrogen source [45]. However, the role of cryptococcal urease in nutrition and metabolism has not been fully explored. Consequently, we tested whether *C*. *neoformans* could use urea as both a nitrogen and carbon source and whether this was urease-dependent. The growth of the three strains were very similar in minimal medium containing glycine as sole nitrogen source and glucose as a carbon source (Fig 8A). When urea was the sole nitrogen source, there was no growth of urease deletion mutant up to 72 h (Fig 8D), consistent to prior results. The addition of ammonium salt partially complemented the growth defect of urease deletion mutation, suggesting that the growth defect was the result of an inability to produce ammonia (Fig 8E). On the other hand, although urea could serve as a nitrogen source, cryptococcal strains were not able to grow when urea was the sole carbon source (Fig 8F). Taken together, our results show that *C*. *neoformans* has an ability to utilize urease to hydrolyze urea into ammonia and use it as nitrogen source, but *C*. *neoformans* cannot utilize urea as carbon source for growth.

### Increased brain invasion by macrophages laden with urease-positive strain

**Fig 9:**
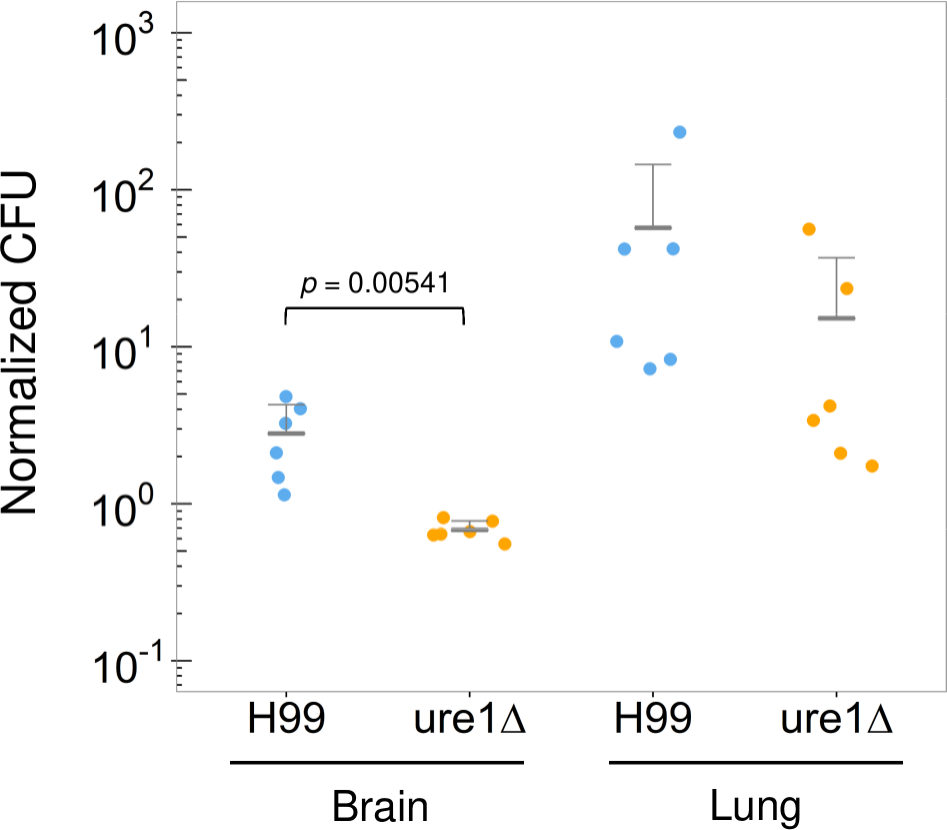
Brain and lung fungal burden in mice injected with macrophages carrying H99 or *ure1*Δ strain. Brain and lung CFU levels in C57BL/6 mice were measured 72 h after retroorbital injection with 1 × 10^7^ cells of macrophages infected with equal number of *C*. *neoformans*. Each dot represents the CFU recovered from an individual mouse and normalized to the inoculum from two independent experiments. Middle bars indicate means and error bars indicate SD. *P* values by Student’s *t* test.

*C*. *neoformans* brain invasion can occur by carriage in macrophages in a Trojan Horse-like mechanism or through transcytosis of endothelial cells [19,23,26,70–77]. Previously, it was reported that urease-negative strains cannot reach the brain from the lungs [44,46]. It is still not clear if this defect was due to ineffective dissemination from the lung or whether this reflected the fact that urease-negative strains are less effective in crossing the BBB [44,46]. Furthermore, it was not clear if Trojan-horse transport inside macrophages was affected by the presence and absence of urease. Since urease retards the intracellular replication, it could promote the coexistence and persistence of *C*. *neoformans* within macrophages and thus increase the chance for dissemination by a Trojan Horse-like mechanism. Alternatively, since *C*. *neoformans* urease also induces non-lytic exocytosis, this could facilitate escape of *C*. *neoformans* from macrophages in the lung or the bloodstream and enhance brain invasion of free yeasts by transcytosis, which is facilitated by urease. Therefore, we hypothesized that macrophages infected with urease-positive strain would be more efficient at the brain invasion than *ure1* infected macrophages. To test this, we injected mice with H99- and *ure1*Δ-infected macrophages and quantified brain and lung fungal burden by CFU at 72 h post-infection. We found lower CFU in the brain of mice infected with BMDM containing *ure1* mutant relative to H99-containing BMDM (Fig 9). In contrast, lung CFU were comparable for mice given macrophages containing either strain, suggesting that brain invasion by Trojan-Horse mechanisms depends on active urease.

## Discussion

Urease is an important virulence factors of *C*. *neoformans* [40]. However, most studies have focused on urease role in brain invasion and its effects on the host immune response. Cryptococcal urease facilitates transmission of *C*. *neoformans* across the blood-brain barrier [44–46] and polarizes the immune system to a Th2 response, which translates into greater fungal burden in lung in mouse model [52]. Yet the effect of urease in macrophage interaction has not been explored despite the fact that the outcome of the interaction *of C*. *neoformans* with macrophages is a key determinant of the outcome of infection [13,78,79]. In this study, we analyzed the role of urease in *C*. *neoformans*-macrophage interactions. Our results provide new insights on how this enzyme can affect the pathogenesis of *Cryptococcus* spp since we show that urease influences the intracellular growth of *C*. *neoformans*, affects non-lytic exocytosis from macrophages, is critical for growth at mammalian physiological pH and confers upon the yeast the potential for using urea as a nitrogen source in nutrition.

Urease can break down urea to produce ammonia, which in turn raises pH. In our study, the measurements of phagolysosomal pH show that cryptococcal urease contributes to buffering acidic pH in the phagolysosome, which is almost certainly a consequence of the hydrolysis of urea. Urea is present in the fetal bovine serum in the culture medium and easily crosses cell membranes. Urea is also a product of macrophages arginase catalysis, which provides *C*. *neoformans* an additional intracellular source of substrate for urease. Moreover, human body fluids normally contain between 2.5 to 7.1 mM urea that is evenly distributed in all body compartments [80–82]. These concentrations make it feasible for *C*. *neoformans* to utilize urea in the host and alter its microenvironmental pH such as phagolysosome. Phagolysosomal alkalization has also been observed with other urease-positive microbes. Mycobacterial urease contributes to the alkalization of the phagolysosomal pH in resting macrophages, but its effect is not sufficient to neutralize the pH in the more acidic phagolysosome of activated macrophages [83]. In contrast, the presence of cryptococcal urease was sufficient to raise pH by an average of 0.4 pH units. *Helicobacter pylori* urease is also known to significantly elevate phagolysosomal pH as well as retard recruitment of Lamp-1, a marker for late endosome and lysosome [50]. However, unlike *H*. *pylori* cryptococcal urease did not affect the LAMP-1 acquisition.

Phagosome acidification is usually considered an important component of the antimicrobial machinery [84], but *C*. *neoformans* grows best in acidic environments while other pathogens’ growth is inhibited by phagolysosome acidification [63,85–87]. Our analysis shows that even inside the mammalian macrophage *C*. *neoformans* grows at a higher rate in pH 5–6. Our results, together with studies of other groups, showed that neutralizing phagolysosomes by treating the macrophages with ammonium chloride inhibited intracellular growth of *C*. *neoformans* [14,63]. Therefore, phagolysosomal acidification does not appear to be an important element of in the control of *C*. *neoformans* by macrophages. However, we show that urease is required for the optimal growth of *C*. *neoformans* at physiological pH. Therefore, we describe a previously unknown role of urease in neutral/alkaline pH tolerance.

The majority of intracellular cryptococcal cells in the urease-positive population did not undergo replication immediately upon entry into macrophages and *ure1* deletion strain initiated replication earlier than wild-type counterparts. This effect was pronounced when the culture medium was supplemented with urea. We attribute this effect to an increase in the phagolysosomal pH from 4.4 to 4.8, a pH that reduced the maximum growth rate of *C*. *neoformans*. Together, urease retards growth in macrophages *in vitro*, and at first glance this is the opposite of what would be expected from a virulence factor. However, this effect has to be considered in the context of the larger picture of cryptococcal pathogenesis. Urease-positive strains still possessed the ability of resisting killing by macrophages and delayed replication could promote a quiescent state intracellularly, which may be associated with persistence of infection [88]. A previous study reported a strong correlation between intracellular replication of *C*. *neoformans* and lysosomal damage due to the increased number of yeasts in macrophages [59]. Indeed, macrophages containing urease-positive cells manifested less phagolysosomal permeabilization than those containing urease-negative cells. Loss of phagolysosomal membrane integrity could be expected to benefit to *C*. *neoformans* by allowing the fungus access to host cytosolic nutrients. However, many bacterial pathogens thrive in vacuoles or phagosome rather than nutrient rich cytoplasm and have developed strategies to maintain phagosomal membrane integrity to avoid immune surveillance pathway and eventually prevent inflammasome-mediated lytic cell death called pyroptosis [89]. A temporary maintenance of membrane integrity could contribute to persistent infections by prolonging the interaction of macrophages and cryptococci. Consistent with this, our results show that when the medium is supplemented with urea, urease-positive cryptococcal cells manifest pronounced growth retardation and cause fewer events of host cell lysis. This correlation suggests that the growth retardation associated with urease mediated alkalization results in fewer yeasts in macrophages, which in turn protects *C*. *neoformans* from humoral immune responses and facilitates persistent infection and dissemination by reducing the likelihood of lytic exocytosis.

The cellular and molecular mechanism of the non-lytic exocytosis is poorly understood. It is a highly choreographed process where both host and pathogen factors are involved [17,24]. Previous studies have shown that cryptococcal capsule and phospholipase B1 contribute to non-lytic exocytosis [17,90]. Host cell membrane protein annexin A2 and signaling kinase ERK5 have also been identified to regulate non-lytic exocytosis [27,91]. Repeated cycles of actin polymerization that form around cryptococci-containing phagosome could potentially inhibit non-lytic exocytosis [92]. Moreover, phagolysosome neutralization by the addition of weak base ammonium chloride and chloroquine increased the frequency of non-lytic exocytosis events [62]. Here we identify urease as new fungal factor that modulates non-lytic exocytosis. Moreover, chemical inhibition of urease enzymatic activity decreased the frequency of non-lytic exocytosis, suggesting that the effect was related to urea hydrolysis. The most likely mechanism is that the pH alteration caused by ureolytic reaction contributes to increasing the frequency of non-lytic exocytosis, which is consistent with our observation that urease activity raised phagolysosomal pH. However, the mechanism on how phagolysosomal pH influences the non-lytic exocytosis remains to be elucidated.

Given that phagolysosomal alkalization can increase non-lytic exocytosis [62], we evaluated whether higher concentrations of urea would increase the frequency of this phenomenon. While increase in exogenous urea increased phagolysosomal pH in those containing urease-positive cells, we also observed increased non-lytic exocytosis in macrophages containing urease-negative cryptococcal cells. Therefore, the effect was not entirely dependent on urease-mediated alkalization, but could also be affected by urea, which can promote the fusion of vesicles with bilayer lipid membrane and thus induce exocytosis [93]. Hence, one possible mechanism for the increased non-lytic exocytosis observed for urease-negative cells is that urea encourages phagolysosome-cell membrane fusion to disgorge yeast cells.

The phagolysosomal pH was measured by conjugating a pH sensitive probe to 18B7 antibody, which binds to cryptococcal capsule, a method that can adapted to any system using antibody-mediated phagocytosis. It is noteworthy that we observed considerable pH variation among individual phagolysosomes. This heterogeneity has been observed in many other studies, and could be caused by many factors [65,94–96], such as differences in the timing of phagocytosis. Despite using centrifugation to enhance the yeast cells contact with macrophages, yeast cells might attach to macrophages but not be engulfed synchronously. A recent study also report that the position of lysosomes determines their pH, such that peripheral lysosomes are less acidic than juxtanuclear ones [65]. Phagolysosomal pH variation could also be attributed to the heterogeneity in protein composition of V-ATPases and NADPH oxidase among individual phagolysosomes [95]. Differences in the metabolic state or age of the infecting cells could also contribute to phagolysosomal pH heterogeneity. In this regard, variability in the infecting *Salmonella* population resulted in heterogeneous macrophage response [97]. Finally, it is possible that the heterogeneity in phagolysosomal pH reflects the outcome of the individual battles between *C*. *neoformans* and macrophages that are fought phagolysosome-to-phagolysosome, such that in some phagolysosomes the microbe gains ascendancy while in others it is suppressed.

*C*. *neoformans* was able to utilize urea as nitrogen source for growth. This metabolic process was entirely urease dependent since the urease deficient strain was unable to grow in medium where urea was the sole nitrogen source. Supplementation with ammonium salt partially rescued the growth of urease-negative strain, suggesting that ammonia generated from ureolytic activity, not urea itself, is the actual nitrogen source for the growth of *C*. *neoformans*. We also showed that urea could not serve as the sole source of carbon for *C*. *neoformans*. Therefore, we propose that *C*. *neoformans* urease activity may also provide an important nutritional function for *in vivo* under nitrogen-limited conditions since urea diffuses easily in tissues and macrophages can generate urea. Of note, two conundrums arise from our studies. Firstly, the partial rescue suggests that cryptococcal urease is further involved in the metabolism of ammonia. Secondly, it remains to be investigated why urease is required for growth at physiological pH. In contrast to other virulence factors such as the capsule, melanin, and phospholipase, a deficiency in urease did not increase the vulnerability of *C*. *neoformans* for amoebae. Hence, the main role of urease in *C*. *neoformans* in its natural environment appears to be nutritional in nature and this enzyme provides an example on how a protein involved in nutrition acquisition can serve a fortuitous role during pathogenesis as a modulator of virulence.

Taken together, we propose the following model for the role of urease in intracellular pathogenesis. Urease secreted by *C*. *neoformans* into phagolysosome hydrolyses urea, releases ammonia and increases the pH of this compartment by approximately half of a pH unit, which is sufficiently to retard the replication of *C*. *neoformans* inside macrophages. The growth retardation in turn leads to fewer macrophages with phagolysosomal membrane permeabilization and fewer host cell lysis. In parallel, *C*. *neoformans* urease induces non-lytic exocytosis events of macrophages. Crossing of the blood brain barrier can be done by yeast cells in a transcytosis event or inside macrophages in a Trojan horse-like mechanism [19,23,26,70–77]. For yeast cells crossing the blood brain barrier alone urease has been shown to promote brain invasion [44,46]. In addition, urease contributes to optimal *C*. *neoformans* growth at physiological pH, which may facilitate the its extracellular growth and dissemination to tissues. Urease can also play a role in cryptococcal nitrogen metabolism by providing a source of nitrogen, and that could support the long-term survival of *C*. *neoformans* in nitrogen-limited conditions such as macrophages. Our observation shows that the presence of urease promotes non-lytic exocytosis, delays intracellular replication, allows for use of an abundant nitrogen source and facilitates growth at mammalian physiological pH. Therefore, urease can affect all of the types of blood-brain barrier crossing mechanisms by providing more extracellular yeasts and increased pH fitness for transcytosis, as well as increasing residence time in macrophages, with the latter promoting macrophage-associated crossings. This supposition was supported by the observation of higher fungal dissemination to the brain in mice injected with macrophage containing urease producing *C*. *neoformans*. Furthermore, it is consistent with the model proposed by others that elevating the frequency of non-lytic exocytosis by altering host cell signaling reduces dissemination, presumably by limiting the opportunity for Trojan horse transport [27]. Overall, we propose that urease helps *C*. *neoformans* to both persist in and exit from macrophages, events that could facilitate the dissemination of the pathogen to brain through macrophage-dependent transport mechanisms. We anticipate that the findings here for *C*. *neoformans* may also be relevant to other urease-positive fungal pathogens such as *Aspergillus fumigatus* and *Histoplasma capsulatum*, two pathogens that modulate phagolysosomal pH and for which acidification is critical for control of infection [87,98,99]. Analysis of urease effects on macrophages is likely to be a fertile area of investigation for the many fungal pathogens that express this enzyme.

## Materials and methods

### Ethics statement

All animal procedures were performed with prior approval from Johns Hopkins University (JHU) Animal Care and Use Committee (IACUC), under approved protocol numbers M015H134. Mice were handled and euthanized with CO_2_ in an appropriate chamber followed by thoracotomy as a secondary means of death in accordance with guidelines on Euthanasia of the American Veterinary Medical Association. JHU is accredited by AAALAC International, in compliance with Animal Welfare Act regulations and Public Health Service (PHS) Policy, and has a PHS Approved Animal Welfare Assurance with the NIH Office of Laboratory Animal Welfare. JHU Animal Welfare Assurance Number is D16-00173 (A3272-01). JHU utilizes the United States Government laws and policies for the utilization and care of vertebrate animals used in testing, research and training guidelines for appropriate animal use in a research and teaching setting.

### *Cryptococcus* strains and growth conditions

The *C*. *neoformans* strains were used in this study are *C*. *neoformans* var. *grubii* serotype A strain H99, *ure1*Δ (derived from H99 and lacking urease) and *ure1*Δ::*URE1* (complemented urease mutant). All the strains were kindly provided by Dr. John Perfect (Duke University, USA) and have been described previously (Cox *et al*. 2000). The urease production phenotype of these strains was validated by using Christensen’s urea agar (2% urea, 1.5% agar, 0.2% KH_2_PO_4_, 0.1% peptone, 0.1% dextrose, 0.5% NaCl, 0.0012% phenol red). Cryptococcal cells were cultivated in Sabouraud dextrose broth with shaking (120 rpm) at 30 °C for overnight (16 h). Heat inactivated or killed *C*. *neoformans* was prepared for various experiments by incubating the cells at 50 °C for 30 min or 4 h.

### Growth kinetics

To study the effect of pH on cryptococcal growth, the yeast cells were grown in minimal medium (15 mM dextrose, 10 mM MgSO_4_, 29.4 mM KH_2_PO_4_, 13 mM glycine, 3 μM thiamine-HCl) buffered with 100 mM citrate buffer (Sodium citrate and citric acid) at various pH ranging from 4.2 to 5.4 at 0.2-pH unit increments. The pH of the medium was measured using an Accumet Basic AB15 pH meter (Thermo fisher Scientific, Waltham, MA). The pH was further verified by use of MColorpHast pH-indicator strips (EMD Millipore, Jaffrey, NH) before and after the growth assay to ensure the pH keep constant throughout the assay.

To study the utilization of urea in *C*. *neoformans*, cells were grown in minimal medium at pH 5.5 with substitution of 7.5 mM urea or ammonium sulfate for glycine as sole nitrogen source and substitution of 7.5 mM urea for dextrose as sole carbon source. Cryptococcal strains were also grown in Sabourand broth to determine their growth and doubling time.

Growth studies were done using a Bioscreen C plate reader (Growth Curves USA) starting the cultures with 10^5^ yeast cells per well in honeycomb plate in different conditions mentioned above at 30 °C and measuring cell density for 72 h.

### Cell culture

Bone-marrow derived macrophages (BMDM) were isolated from the marrow of hind leg bones of 5- to 8-wk-old C57BL-6 female mice (Jackson Laboratories, Bar Harbor, ME. For the differentiation, cells were seeded in 100 mm TC-treated cell culture dishes (Corning, Corning, NY) in Dulbecco’s Modified Eagle medium (DMEM; Corning) with 20% L-929 cell-conditioned medium, 10% FBS (Atlanta Biologicals, Flowery Branch, GA), 2mM Glutamax (Gibco, Gaithersburg MD), 1% nonessential amino acid (Cellgro, Manassas, VA), 1% HEPES buffer (Corning), 1% penicillin-streptomycin (Corning) and 0.1% 2-mercaptoethanol (Gibco) for 6–7 days at 37 °C with 9.5% CO_2_. Fresh media in 3 ml were supplemented on day 3 and the medium were replaced on day 6. Differentiated BMDM were used for experiments within 5 days after completed differentiation. Urea in 9 mM or 50 mM were supplemented in the medium during infection of macrophages with *C*. *neoformans* in some of the experiments. The amount of urea inside macrophages were measured using urea colorimetric assay (Sigma-Aldrich, St. Louis, MO) according to the manufacturer’s instruction.

J774.16 cells, which were obtained from the American Type Culture Collection (ATCC), is a murine (BALB c, haplotype H-2d) macrophage-like cell line derived from a reticulum sarcoma. J774.16 cells were maintained in DMEM with 10% NCTC109 medium (Gibco), 10% FBS, 1% nonessential amino acid, 1% penicillin-streptomycin at 37 °C with 9.5% CO_2_.

*Acanthamoeba castellanii* strain 30234 was obtained from the American Type Culture Collection (ATCC) was maintained in peptone-yeast extract-glucose (PYG) broth (ATCC medium 712) at 25 °C according to instructions from ATCC.

### Urease inhibition and activity detection

*C*. *neoformans* strains were grown overnight in Sabouraud broth, and diluted into 5 × 10^7^ cells in 2 ml of rapid urea broth (RUH) developed by Roberts [100] and adapted by Kwon-Chung [101]. Different concentrations (1.25–40 mM) of urease inhibitor acetohydroxamic acid (AHA) were added. Cells were incubated at 37 °C for 7 and 24 h. In parallel, H99 and *ure1*Δ strains were grown without AHA as positive and negative controls. After incubation, cells were collected by centrifugation and 200 μl of supernatant were transferred to 96-well plate. The absorbance of the supernatant was measured at 570 nm using EMax Plus microplate reader (Molecular Devices). The assay was performed in duplicate for each time interval.

### Microscopy and time-lapse imaging

BMDM were seeded (5 × 10^4^ cells/well) on poly-D-lysine coated coverslip bottom MatTek petri dishes with 14mm microwell (MatTek Brand Corporation) in medium containing 0.5 μg/ml lipopolysaccharide (LPS; Sigma-Aldrich), 100 U/ml gamma interferon (IFN-γ; Roche). Cells were then incubated at 37 °C with 9.5% CO_2_ overnight. On the following day, macrophages were infected with cryptococcal cells (1.5 × 10^5^ cells/well) in the presence of 10 μg/ml monoclonal antibody (Mab) 18B7. After 2 h incubation to allow phagocytosis, culture was washed five times with fresh medium to remove extracellular cryptococcal cells. Images were taken every 4 min for 24 h using a Zeiss Axiovert 200M inverted microscope with a 10x phase objective in an enclosed chamber under conditions of 9.5% CO_2_ and 37 °C. For some of the experiments, 9 mM urea was added into the BMDM culture for overnight incubation, or both BMDM and cryptococcal cells were pretreated with 20 mM ammonium chloride or 5 mM acetohydroxamic acid (AHA) for 30 min before phagocytosis. The chemicals were also present during both the 2 h incubation to permit phagocytosis and the 24 h incubation during time-lapse imaging.

### Phagolysosomal pH measurement

Phagolysosomal pH was measured using ratiometric fluorescence imaging involving the use of pH-sensitive probe Oregon green 488. Oregon green 488 was first conjugated to monoclonal antibody 18B7 using Oregon Green 488 Protein Labeling Kit (Molecular Probes, Eugene, OR). The Oregon Green 488 dye has a succinimidyl ester moiety that reacts with primary amines of proteins to form stable dye-protein conjugates. The labeling procedure is according to the manufacture’s instruction. BMDM were plated (1.25 × 10^5^ cells/well) on 24-well plate with 12 mm circular coverslip. Cells were cultured with completed BMEM medium containing 0.5 μg/ml LPS and 100 U/ml IFN-γ; as well as supplemented with or without urea at 9 mM or 50 mM, and then incubated at 37 °C with 9.5% CO_2_ overnight. Prior to infection, macrophages were placed at 4 °C for 15 min. In the meanwhile, live, heat inactivated, heat killed cryptococcal strains or anti-mouse IgG coated polystyrene bead (3.75 × 10^6^ cells or beads/ml) were incubated with 10 μg/ml Oregon green conjugated 18B7 Ab for 15 min. Macrophages were then infected with Oregon green conjugated 18B7-opsonized samples in 3.75 × 10^5^ cells or beads per well. Cells were centrifuged immediately at 1200 rpm for 1 min and culture were incubated at 37 °C for 10 min to allow phagocytosis. Extracellular cryptococcal cells or beads were removed by washing three times with fresh medium. Samples on coverslip were collected at 1, 2, 3, 4 h after phagocytosis by washing twice with pre-warmed HBSS and placing upside down on MatTek petri dish (MatTek, Ashland, MA) with HBSS in the microwell. Images were taken by using Olympus AX70 microscopy (Olympus, Center Valley, PA) with objective 40x at dual excitation 440 nm and 488 nm, and emission 520 nm. Images were analyzed using MetaFluor Fluorescence Ratio Imaging Software (Molecular Devices, Downingtown, PA). Relative phagolysosomal pH was determined based on the ratio of 488 nm/440 nm. The relative pH was converted to absolute pH by obtaining the standard curve in which the images are taken as above but intracellular pH of macrophages was equilibrated by adding 10 μM nigericin in pH buffer (140 mM KCl, 1 mM MgCl_2_, 1 mM CaCl_2_, 5 mM glucose, and appropriate buffer ≤ pH 5.0: acetate-acetic acid; pH 5.5–6.5: MES; ≥pH 7.0: HEPES. Desired pH values were adjusted H using either 1M KOH or 1M HCl). Buffers were used at pH 3–7.5 using 0.5-pH unit increments.

### Phagolysosomal membrane permeabilization assay

J774.16 cells were plated (2 × 10^6^ cells/well) on 6-well plate with completed DMEM containing 0.5 μg/ml LPS, 100 U/ml IFN-γ, with or without urea at 9 mM, and incubated at 37 °C with 9.5% CO_2_ overnight. On the following day, cryptococcal cells were stained with 0.0015% Uvitex 2B (Polysciences) for 1 min and wash once with medium. Macrophages were infected with Uvitex 2B-stained cryptococcal cells (1 × 10^6^ cells/well) in the presence of 10 μg/ml 18B7 for 24 h. After 24 h infection, Lysotracker deep red (Thermo Fisher Scientific) at 1 nM is added to the culture and incubated for 1 h. Cells were harvested from plates and washed once with HBSS. Anti-mouse CD11b-PE (1:1000) (M1/70, eBioscience, San Diego, CA) was added and incubated for 5 min and washed once with HBSS. SYTOX and F2N12S (Thermo Fisher scientific) were added 5 min before flow cytometry analysis. Single color and fluorescence minus one (FMO) controls were used for fluorescence spectral compensation and gating. Flow cytometry analysis were performed by LSRII (BD Biosciences, San Jose, CA). Data were analyzed using FlowJo software (Ashlan, OR).

### Intracellular survival assay

BMDM cells (5 × 10^4^ cells/well) were seeded in 96-well plates with BMDM containing 0.5 μg/ml LPS and 100 U/ml IFN-γ for overnight. To initiate the phagocytosis, *C*. *neoformans* with 1.5 × 10^4^ cells in the presence of 10 μg/ml 18B7 mAb were added in each well of BMDM culture. The culture plates were centrifuged at 1200 rpm for 1 min to settle yeast cells on the monolayer of macrophage culture. After 2 h infection, phagocytized cryptococcal cells were released by lysing the macrophages with sterilized water. The lysates were serially diluted, plated onto Sabouraud agar and incubated at 30 °C for 48 h for colony form unit (CFU) determination. This experiment was performed in triplicates for each strain.

### Amoebae assay

The survival of *C*. *neoformans* in amoebae culture was performed as described previously [55]. Briefly, *A*. *castellanii* were washed twice with DPBS (Corning) and diluted in DPBS to appropriate density. *A*. *casterllanii* cells (1 × 10^4^ cells/well) were added to 96-well plates and allowed to adhere for 1 h at 25 °C. *C*. *neoformans* cells were washed twice with DPBS and diluted in DPBS to appropriate density. Fungal cells (1 × 10^4^) were added to wells containing amoebae or control wells containing DPBS alone, and the plates were incubated at 25 °C. At 0, 24, and 48 h, the amoebae were lysed by pulling the culture through a 27-gauge syringe needles five to seven times. The lysates were serially diluted, plated onto Sabouraud agar and incubated at 30 °C for 48 h for colony form unit (CFU) determination. Two different conditions were tested with DPBS supplemented with or without 7.5 mM urea. Two biological independent experiments were performed for each strain and condition. Viability of *A*. *castellanii* was also determined under the same conditions and time intervals by adding 1:80 dilution of Trypan Blue stain. The percentage of dead amoebae was determined by counting the number of Trypan Blue stained cells per total cell number counted. Minimal of 100 cells were counted. Control wells contain *A*. *castellanii* without *C*. *neoformans*. Two biological independent experiments were performed for each strain and condition.

### Capsule size measurement

After 16 h infection, macrophage-internalized cryptocooccal strains were released by lysing the host cell with sterile water. Cryptococcal cells were washed twice with PBS. The capsule was visualized by India ink negative staining by mixing cell samples with equal volume of India ink on glass slides and spreading the smear evenly with coverslips. The images with a minimum 100 randomly chosen cells was taken by using Olympus AX70 microscopy with 100x oil objective using the QCapture Suite V2.46 software (QImaging, Surrey, Canada). The areas of cell body and whole cell (cell body plus capsule) were measured using image J software. The capsule area was calculated by subtracting the area of whole cell from that of cell body. Three biological independent experiments were performed for each strain.

### Immunofluorescence staining

BMDM (1.5 × 10^5^ cells) were seeded on 12 mm circular coverslip in 24-well plate with completed BMDM containing 0.5 μg/ml LPS and 100 U/ml IFN-γ for overnight. *C*. *neoformans* with 1.5 × 10^5^ cells in the presence of 10 μg/ml Alexa Fluor 568 conjugated 18B7 mAb were then added into BMDM culture. The culture plates were centrifuged at 1200 rpm for 1 min to settle yeast cells on the monolayer of macrophage culture. After 10 min, 30 min, 1 h and 2 h infection, cells were fixed with 4% paraformaldehyde (Electron Microscopy Sciences, Hatfield, PA), followed by permeabilization with 0.3% Triton X-100 in PBS for 5 min and incubated with blocking solution containing 3% bovine serum albumin and 1:250 dilution of purified rat anti-mouse CD16/CD32 (Mouse BD FC Block; BD Pharmingen, San Diego, CA) for 45 min. Cells were next incubated with 1:50 dilution of Alex Fluor 488 conjugated anti-mouse Lamp-1 (rat IgG_2a_ monoclonal antibody 1D4B; Santa Cruz Biotechnology Inc., Santa Cruz, CA) at 4 °C overnight and then washed three times with 1×PBS for 5 min each time. Coverslips were mounted using ProLong Gold Antifade Mountant (Thermo Fisher Scientific) and cured for 24 h at room temperature. The images with a minimum 100 randomly chosen cells was acquired by Zeiss Axiovert 200M inverted microscope with a 40x objective. Z-stacks were taken at 1 μm intervals through entire macrophage. Phagosome-lysosome fusion was considered to take place when there is co-localization of cryptococcal capsule (Alexa Fluor 568) and Lamp-1 (Alexa Fluor 488). Two biological independent experiments were performed for each strain.

### Nitrite measurement

BMDM cells (1 × 10^5^ cells/well) were activated by LPS (0.5 μg/ml) and IFN-γ (100 U/ml) for overnight in 96-well plates. To initiate infection, *C*. *neoformans* (1 × 10^5^ cells) with 10 μg/ml 18B7 mAb were added and settled down on macrophage monolayer culture using centrifugation at 1200 rpm for 1 min. After 24 h infection, culture supernatant in 100 μl was collected and equal volume of Griess reagent (1: 1 ratio of 0.1% naphtylethylenediamine dihydrochloride and 1% sulfanilamide in 5% H_3_PO_4_) was added. The mixture was incubated in the dark for 10 min at room temperature. The absorbance of the mixture was measured at 562 using EMax Plus microplate reader (Molecular Devices). Nitrite concentration was determined from a standard curve constructed with 0 μM–50 μM sodium nitrite. Two biological independent experiments were performed for each strain and condition.

### Mice infection

Animal studies were performed using 6 to 8-week-old female C57BL/6 mice. Cryptococcal strains were grown for 2 days at 37 °C with shaking at 180 rpm in Sabouraud broth. Cells were washed with PBS and resuspended to 1 × 10^7^ cells/ml in BMDM medium. Cryptococcal cells in 1 ml was added to BMDM in triplicates (1 × 10^7^ cells per replicate) together with opsonizing 18B7 (final concentration of 10 μg/ml). After 1 h incubation to allow phagocytosis, extracellular cryptococcal cells were washed with HBSS and infected BMDM were detached with CellStripper (Corning), collected by centrifugation. The infected BMDM were then resuspended in USP grade sterile saline solution (BD, Franklin Lakes, NJ). Each mouse was injected i.v. with 200 μl of cell suspension. Mice were anesthetized with 2% isoflurane anesthesia followed by retroorbital injection of BMDM suspension, according to standard procedures [102]. Infected BMDM were lysed with sterilized water and cell lysis were plated in YPD plates to confirm inoculum CFU. After 72 h post-injection, mice were euthanized, lung and brain were isolated and homogenized by passing through a 100 μm filter. Homogenates were plated onto YPD agar for CFU enumeration. Three mice per strain per experiment were studied and two independent biological experiments were performed.

### Statistical analysis

Pairwise comparisons depicted in Fig 6 are Mann-Whitney *U* test with both wild-type and *ure1* complement strains comparing to *ure1* deletion mutant. One-way ANOVA, followed by Tukey’s multiple-comparison test was used to evaluate the statistical parameters of characteristic growth values. For categorical data, Fisher’s exact test was used when sample sizes were less than 1000 or chi-square test was used when sample sizes were larger than 1000. All other continuous data were analyzed by Student’s *t* test.

## Supporting information

S1 Fig (A) Urease has no effect on phagocytosis by murine macrophages. BMDM was incubated with cryptococcal urease-positive strain (H99 or *ure1*Δ::*URE1*) and urease-negative strain (*ure1*Δ) for 2 h to allow phagocytosis. The *C*. *neoformans*/macrophage ratio was 3:1. The phagocytic index was determined by the number of internalized cryptococcal cells per 100 macrophages. Each strain was tested at least five times independently for over 600 macrophages. Error bars represent 95% confidence interval of the mean. *P* > 0.05 by Fisher’s exact test. (B) Urease has no effect on the recruitment of lamp-1 to phagosome. BMDM were infected with H99, *ure1*Δ or *ure1*Δ::*URE1* strain for indicated times. Cells were fixed and stained with lamp-1 antibody, and processed for imaging. Percentage of phagosomes which acquire lamp-1 were shown. Two independent biological experiments were performed. *P* > 0.05 by Student’s *t* test. (C) Urease does not affect the level of NO_2_^-^ generation by BMDM. BMDM were infected with H99, *ure1*Δ or *ure1*Δ::*URE1* strain for 24 h. The level of NO_2_^-^ in the culture supernatant were determined by Griess reaction colorimetric nitrite assay. The data are presented as mean ± SD from triplicate observations. Comparable result was obtained from additional independent experiment. *P* > 0.05 by Student’s *t* test. (D) Urease does not affect the survival of *C*. *neoformans* inside macrophage. The survival of cryptococcal strains was determined by colony form unit (CFU) after 0 and 2 h phagocytosis. The percentage of survival was calculated by normalizing the CFU value of 2 h infection to that of time zero. Data represent the mean of three technical replicates per biological sample and error bars are SD. Comparable result was obtained from additional independent experiment. *P* > 0.05 by Student’s *t* test.
(TIF)

S2 Fig (A) The morphologies of BMDM after treatment with indicated concentration of AHA for 24 h. (B) The percentage of dead BMDM was determined by counting the number of Trypan Blue staining cells per total cell number counted. Three independent biological experiments were performed. Error bars are SD. (C) The urease activity of cryptococcal cells in different concentrations of AHA were detected by using rapid urea broth (RUH) method. Error bars represent SD. The assay was performed in duplicate for each time point.
(TIF)

S3 Fig The presence of urease does not affect the size of capsule during macrophage infection. Macrophage-internalized cryptococcal strains were released after 16 h infection and their capsule was visualized by India ink negative staining. The capsule area was calculated by subtracting the area of whole cell from that of cell body. Each dot represents the capsule area of each cell. Violin plot displays the probability density of dataset with minimal of 100 cells with means (middle bar) and error bars. Error bars are SD. Comparable result was obtained from additional independent experiment. *P* > 0.05 by Student’s *t* test.
(TIF)

S4 Fig (A) Standard curve for BMDM laden with Oregon green labeled *C*. *neoformans* fluorescence excitation ratio (488ex/440ex,: 520em). (B) Summary plot for replicates on pH measurement of phagolysosome which is loaded with beads. Each dot represents the mean of phagolysosomal pH measured in each replicate. Error bars are SD (C) Additional biological replicates of pH measurement on phagolysosomes, which contain H99, *ure1*Δ, *ure1*Δ::*URE1* strains. (D-E) Summary plot of the means and SD for replicates for replicates on pH measurement of phagolysosome. Each dot represents the mean of phagolysosomal pH measured in each replicate.
(TIF)

S5 Fig (A) The amount of urea in macrophages under the conditions tested. Macrophages were cultured in the medium either with no urea or 9 mM urea supplement for 4 h. Cells were lysed and the amount of urea of lysate were determined by urea colorimetric assay. *P* value by Student’s *t* test. (B) Summary plot of the replicates on phagolysosomal pH measurement under urea supplementation (9 mM and 50 mM). Each dot represents the mean of phagolysosomal pH measured in each replicate. Error bars are SD.
(TIF)
