# Supplementary figures and images for: Cryptococcus neoformans urease affects the outcome of intracellular pathogenesis by modulating phagolysosomal pH
Source: PLoS Pathog. 2018 Jun 15;14(6):e1007144. doi: 10.1371/journal.ppat.1007144 (PMC6021110; doi:10.1371/journal.ppat.1007144)

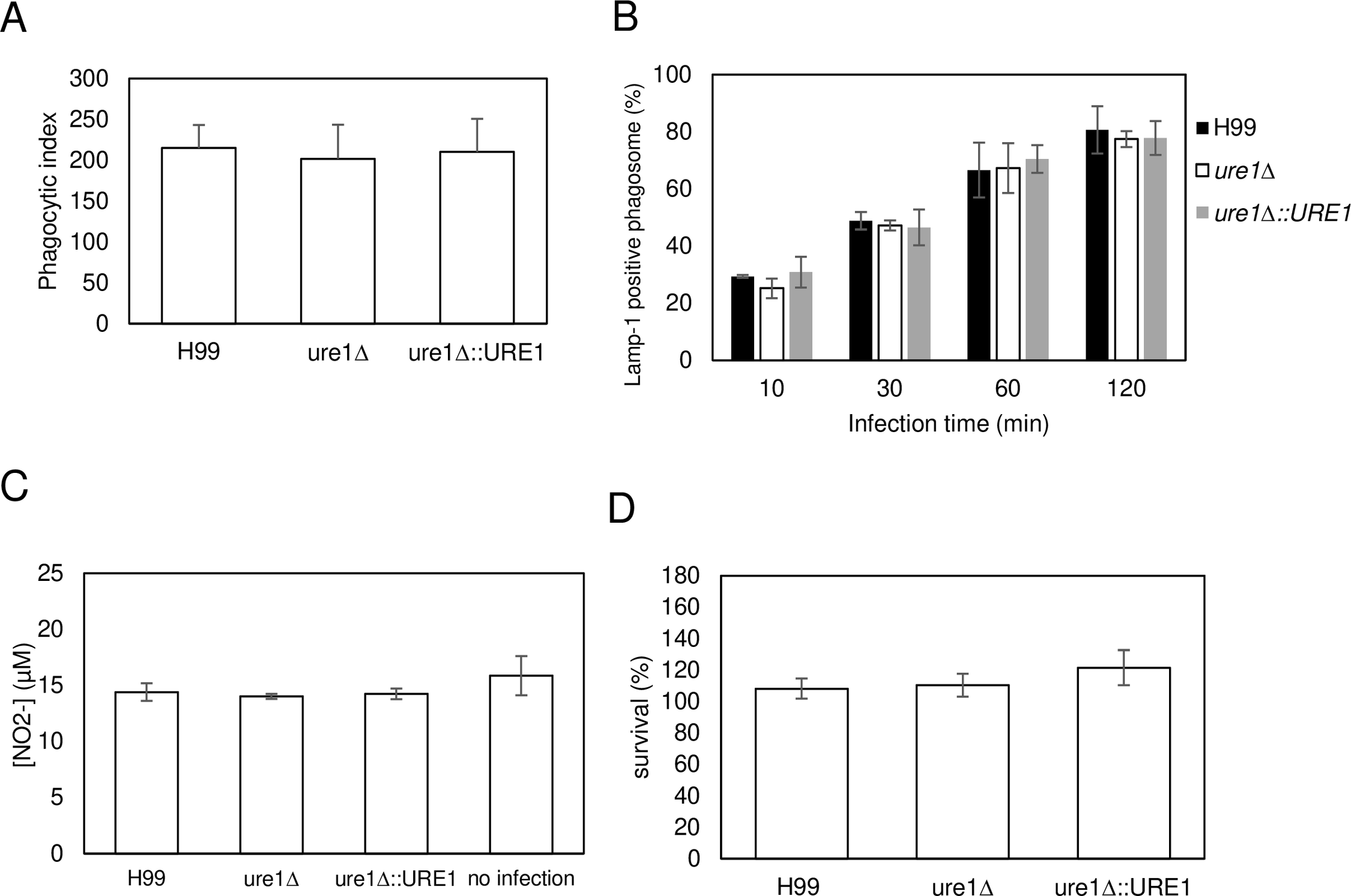

Supplement: S1 Fig — (A) Urease has no effect on phagocytosis by murine macrophages. BMDM was incubated with cryptococcal urease-positive strain (H99 or ure1Δ::URE1) and urease-negative strain (ure1Δ) for 2 h to allow phagocytosis. The C. neoformans/macrophage ratio was 3:1. The phagocytic index was determined by the number of internalized cryptococcal cells per 100 macrophages. Each strain was tested at least five times independently for over 600 macrophages. Error bars represent 95% confidence interval of the mean. P > 0.05 by Fisher’s exact test. (B) Urease has no effect on the recruitment of lamp-1 to phagosome. BMDM were infected with H99, ure1Δ or ure1Δ::URE1 strain for indicated times. Cells were fixed and stained with lamp-1 antibody, and processed for imaging. Percentage of phagosomes which acquire lamp-1 were shown. Two independent biological experiments were performed. P > 0.05 by Student’s t test. (C) Urease does not affect the level of NO2- generation by BMDM. BMDM were infected with H99, ure1Δ or ure1Δ::URE1 strain for 24 h. The level of NO2- in the culture supernatant were determined by Griess reaction colorimetric nitrite assay. The data are presented as mean ± SD from triplicate observations. Comparable result was obtained from additional independent experiment. P > 0.05 by Student’s t test. (D) Urease does not affect the survival of C. neoformans inside macrophage. The survival of cryptococcal strains was determined by colony form unit (CFU) after 0 and 2 h phagocytosis. The percentage of survival was calculated by normalizing the CFU value of 2 h infection to that of time zero. Data represent the mean of three technical replicates per biological sample and error bars are SD. Comparable result was obtained from additional independent experiment. P > 0.05 by Student’s t test. (TIF) [file ppat.1007144.s001.tif]

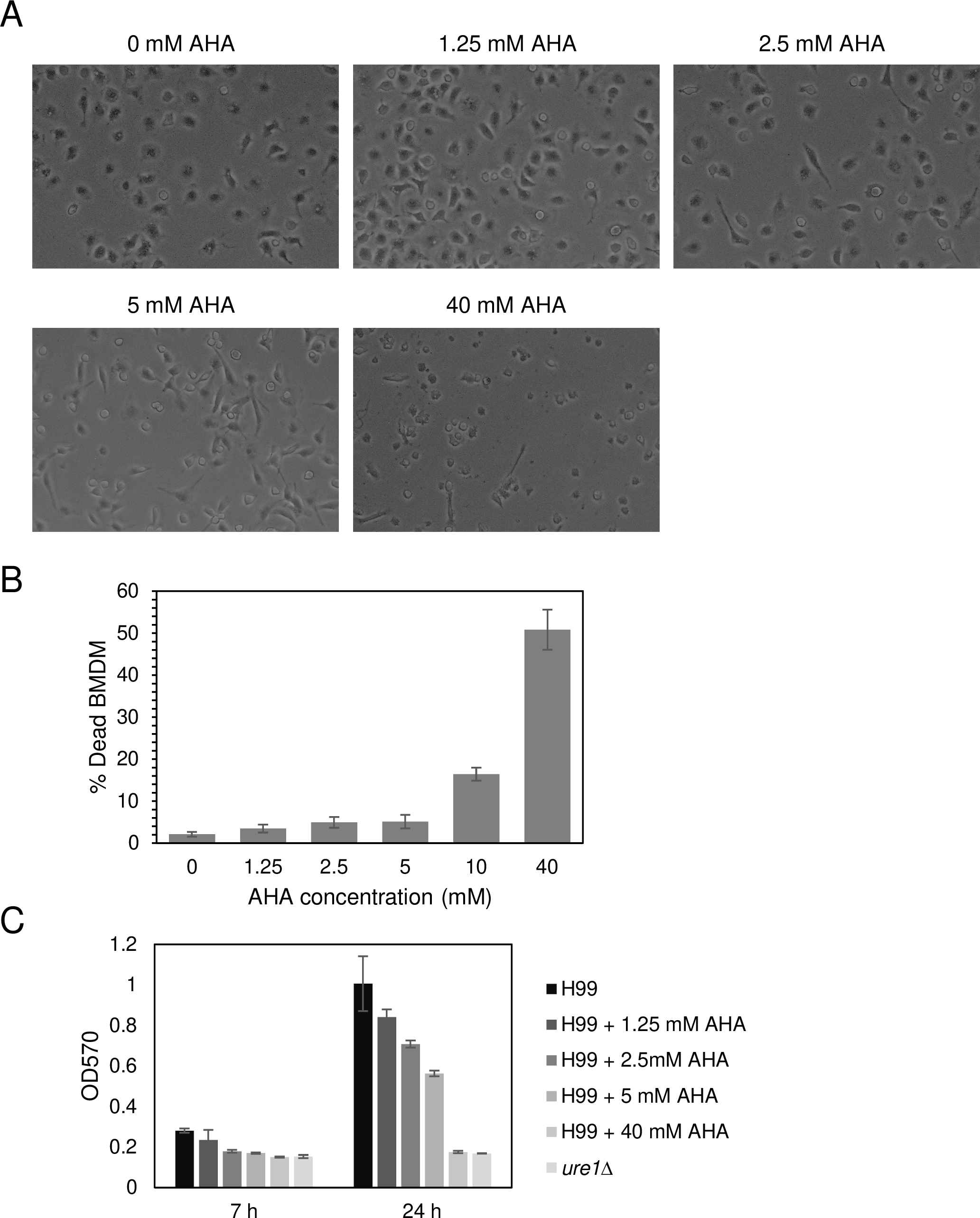

Supplement: S2 Fig — (A) The morphologies of BMDM after treatment with indicated concentration of AHA for 24 h. (B) The percentage of dead BMDM was determined by counting the number of Trypan Blue staining cells per total cell number counted. Three independent biological experiments were performed. Error bars are SD. (C) The urease activity of cryptococcal cells in different concentrations of AHA were detected by using rapid urea broth (RUH) method. Error bars represent SD. The assay was performed in duplicate for each time point. (TIF) [file ppat.1007144.s002.tif]

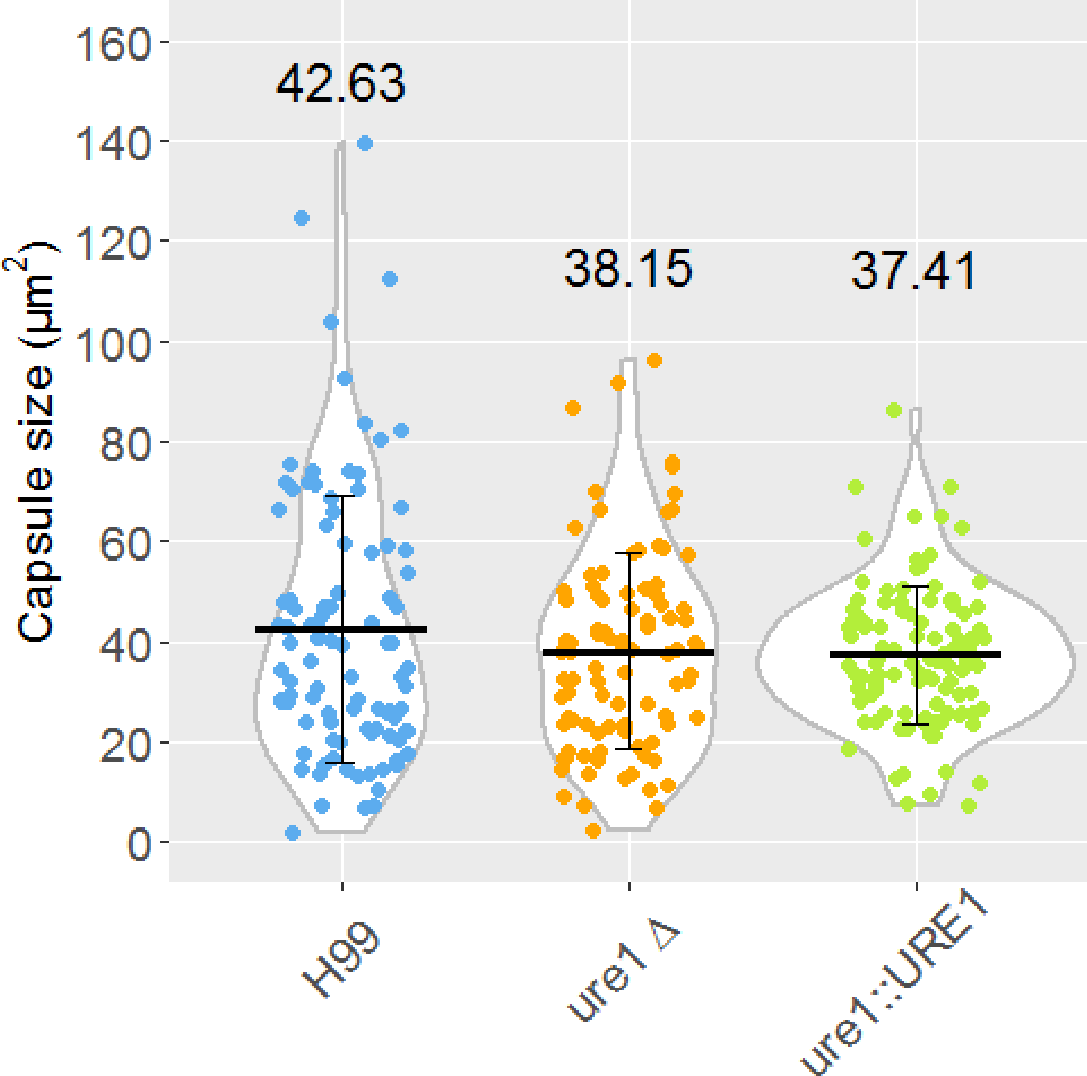

Supplement: S3 Fig — Macrophage-internalized cryptococcal strains were released after 16 h infection and their capsule was visualized by India ink negative staining. The capsule area was calculated by subtracting the area of whole cell from that of cell body. Each dot represents the capsule area of each cell. Violin plot displays the probability density of dataset with minimal of 100 cells with means (middle bar) and error bars. Error bars are SD. Comparable result was obtained from additional independent experiment. P > 0.05 by Student’s t test. (TIF) [file ppat.1007144.s003.tif]

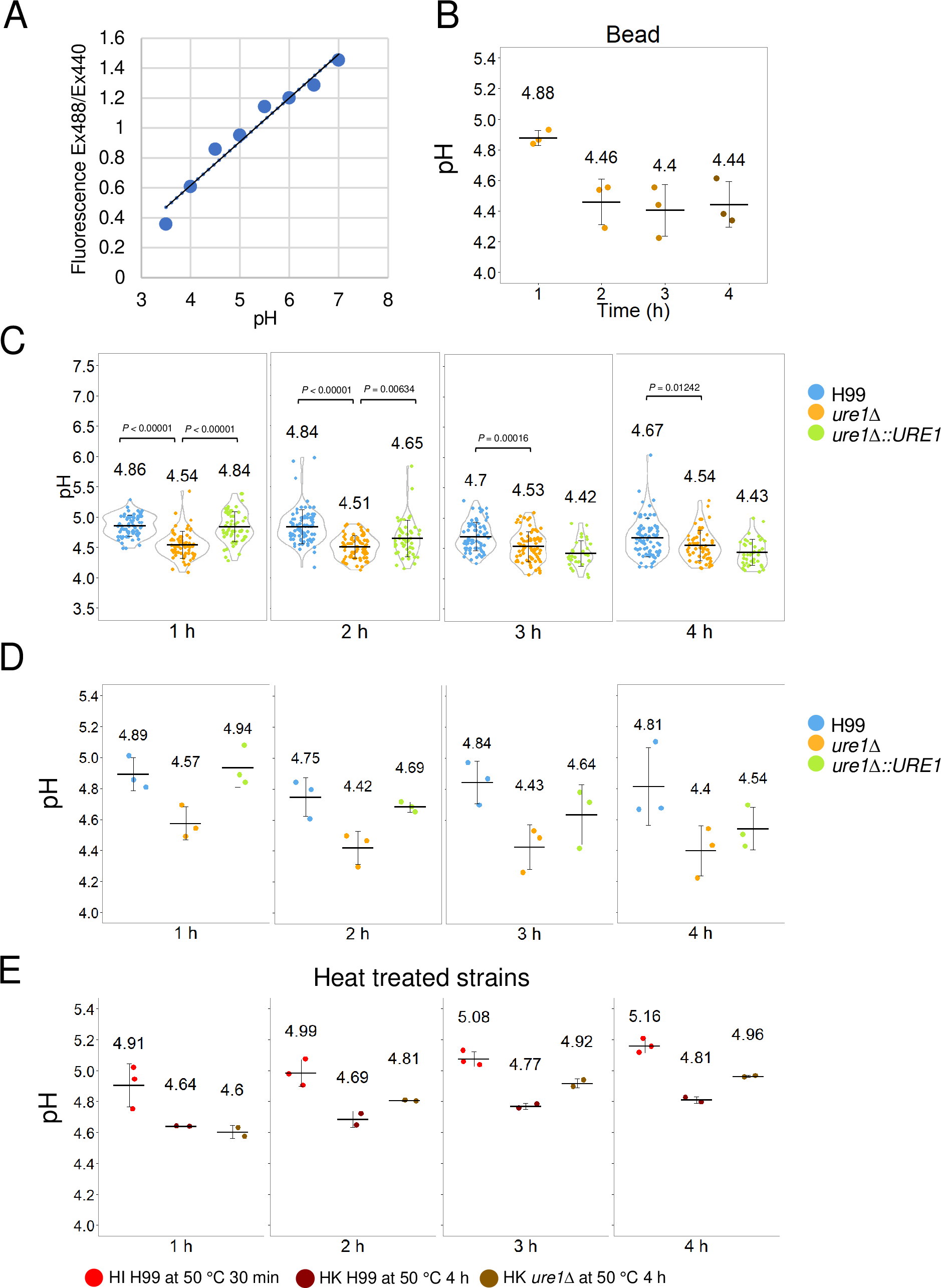

Supplement: S4 Fig — (A) Standard curve for BMDM laden with Oregon green labeled C. neoformans fluorescence excitation ratio (488ex/440ex,: 520em). (B) Summary plot for replicates on pH measurement of phagolysosome which is loaded with beads. Each dot represents the mean of phagolysosomal pH measured in each replicate. Error bars are SD (C) Additional biological replicates of pH measurement on phagolysosomes, which contain H99, ure1Δ, ure1Δ::URE1 strains. (D-E) Summary plot of the means and SD for replicates for replicates on pH measurement of phagolysosome. Each dot represents the mean of phagolysosomal pH measured in each replicate. (TIF) [file ppat.1007144.s004.tif]

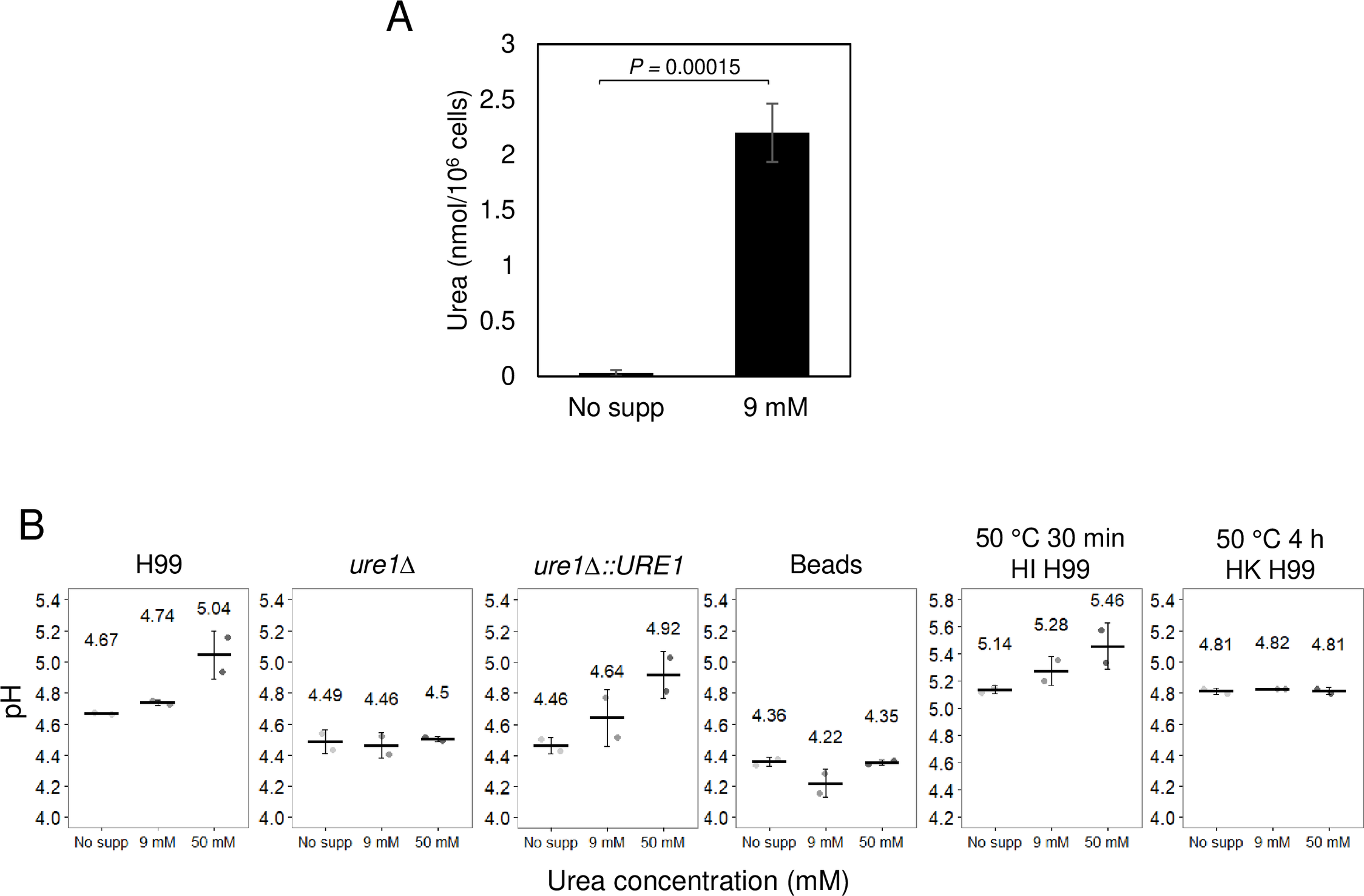

Supplement: S5 Fig — (A) The amount of urea in macrophages under the conditions tested. Macrophages were cultured in the medium either with no urea or 9 mM urea supplement for 4 h. Cells were lysed and the amount of urea of lysate were determined by urea colorimetric assay. P value by Student’s t test. (B) Summary plot of the replicates on phagolysosomal pH measurement under urea supplementation (9 mM and 50 mM). Each dot represents the mean of phagolysosomal pH measured in each replicate. Error bars are SD. (TIF) [file ppat.1007144.s005.tif]
